# Online analysis of microendoscopic 1-photon calcium imaging data streams

**DOI:** 10.1371/journal.pcbi.1008565

**Published:** 2021-01-28

**Authors:** Johannes Friedrich, Andrea Giovannucci, Eftychios A. Pnevmatikakis

**Affiliations:** 1 Flatiron Institute, Simons Foundation, New York, New York, United States of America; 2 Joint Department of Biomedical Engineering, University of North Carolina at Chapel Hill and North Carolina State University; and UNC Neuroscience Center, University of North Carolina at Chapel Hill, Chapel Hill, North Carolina, United States of America; University College London, UNITED KINGDOM

## Abstract

*In vivo* calcium imaging through microendoscopic lenses enables imaging of neuronal populations deep within the brains of freely moving animals. Previously, a constrained matrix factorization approach (CNMF-E) has been suggested to extract single-neuronal activity from microendoscopic data. However, this approach relies on offline batch processing of the entire video data and is demanding both in terms of computing and memory requirements. These drawbacks prevent its applicability to the analysis of large datasets and closed-loop experimental settings. Here we address both issues by introducing two different online algorithms for extracting neuronal activity from streaming microendoscopic data. Our first algorithm, OnACID-E, presents an online adaptation of the CNMF-E algorithm, which dramatically reduces its memory and computation requirements. Our second algorithm proposes a convolution-based background model for microendoscopic data that enables even faster (real time) processing. Our approach is modular and can be combined with existing online motion artifact correction and activity deconvolution methods to provide a highly scalable pipeline for microendoscopic data analysis. We apply our algorithms on four previously published typical experimental datasets and show that they yield similar high-quality results as the popular offline approach, but outperform it with regard to computing time and memory requirements. They can be used instead of CNMF-E to process pre-recorded data with boosted speeds and dramatically reduced memory requirements. Further, they newly enable online analysis of live-streaming data even on a laptop.

## Author summary

Calcium imaging methods enable researchers to measure the activity of genetically-targeted large-scale neuronal subpopulations. Whereas previous methods required the specimen to be stable, e.g. anesthetized or head-fixed, new brain imaging techniques using microendoscopic lenses and miniaturized microscopes have enabled deep brain imaging in freely moving mice. However, the very large background fluctuations, the inevitable movements and distortions of imaging field, and the extensive spatial overlaps of fluorescent signals complicate the goal of efficiently extracting accurate estimates of neural activity from the observed video data. Further, current activity extraction methods are computationally expensive due to the complex background model and are typically applied to imaging data long after the experiment is complete. Moreover, in some scenarios it is necessary to perform experiments in real-time and closed-loop—analyzing data on-the-fly to guide the next experimental steps or to control feedback –, and this calls for new methods for accurate real-time processing. Here we address both issues by adapting a popular extraction method to operate online and extend it to utilize GPU hardware that enables real time processing. Our algorithms yield similar high-quality results as the original offline approach, but outperform it with regard to computing time and memory requirements. Our results enable faster and scalable analysis, and open the door to new closed-loop experiments in deep brain areas and on freely-moving preparations. Our algorithms can be used for newly enabled real-time analysis of streaming data, as well as swapped in directly to replace the computationally costly offline approach.

## Introduction

In vivo calcium imaging of activities from large neural populations at single cell resolution has become a widely used technique among experimental neuroscientists. Recent advances in optical imaging technology using a 1-photon-based miniscope and a microendoscopic lens have enabled in vivo calcium imaging studies of neural activities in freely behaving animals [1–4]. However, this data typically displays large, highly structured background fluctuations due to fluorescence contributions from neurons outside the focal plane, arising from the large integration volume of one photon microscopy. To obtain a robust approach for extracting single-neuronal signals from microendoscopic data the constrained nonnegative matrix factorization (CNMF, [5]) approach has been extended to leverage a more accurate and flexible spatio-temporal background model, able to capture the properties of the strong background signal (CNMF-E, [6]). This prevalent algorithm (see [7] for an alternative proposal) has been widely used to study neural circuits in cortical and subcortical brain areas, e.g. prefrontal cortex (PFC, [8]) and hippocampus [2, 9], as well as previously inaccessible deep brain areas, such as striatum [10, 11], amygdala [12], substantia nigra pars compacta (SNc) [13], nucleus accumbens [14], dorsolateral septum [15], parabrachial nucleus [16], and other brain regions.

A concomitant feature of the refined background model in CNMF-E is its high computational and memory cost. Although the data can be processed by splitting and processing the FOV in smaller patches to exploit a time/memory tradeoff [17], this strategy requires significant time resources and does not scale to longer recordings. Further, CNMF-E is applied to imaging data after the experiment is complete. However, in many cases we would prefer to run closed-loop experiments—analyzing data on-the-fly to guide the next experimental steps or to control feedback [18–20]—and this requires new methods for accurate real-time processing.

Online (and real time) analysis of calcium imaging data has been proposed with the OnACID algorithm [21]. The algorithm combines the online NMF algorithm of [22], the CNMF source extraction algorithm of [5], and the near-online deconvolution algorithm of [23], to provide an automated pipeline that can discover and track the activity of hundreds of cells in real time, albeit only for 2-photon or light-sheet imaging data.

In this paper, we present two algorithms for the online analysis of microendoscopic 1-photon calcium imaging data streams. Our first algorithm (OnACID-E), extends [21] by incorporating the background model and neuron detection method of CNMF-E [6] and adapting them to an online setup. Our second approach proposes a lower dimensional background model by introducing parameter sharing through a convolutional structure and combines it with the online 2-photon processing of [21]. In either approach, every frame is processed in four sequential steps: i) The frame is registered against the previous background-corrected denoised frame to correct for motion artifacts. ii) The fluorescence activity of the already detected sources is tracked. iii) Newly appearing neurons and processes are detected and incorporated to the set of existing sources. iv) The fluorescence trace of each source is denoised and deconvolved to provide an estimate of the underlying spiking activity.

Our resulting framework is highly scalable with minimal memory requirements, as it processes the data in streaming mode (one frame at a time), while keeping in memory a set of low dimensional sufficient statistics and a small minibatch of the most recent data frames. Moreover, it results in faster processing that can reach real time speeds for common experimental scenarios. We apply our framework to typical mouse *in vivo* microendoscopic 1p datasets; our algorithm can find and track hundreds of neurons faster than real-time, and outperforms the CNMF-E algorithm of [6] with regard to computing time and memory requirements while maintaining the same high quality of the results. We also provide a Python implementation of our methods as part of the CaImAn package [17].

## Methods

This section is organized as follows. The first subsection briefly reviews the modeling assumptions of CNMF-E for microendoscope data. In the second subsection, we derive an online method to fit this model, thus enabling the processing of 1-photon endoscopic data streams (OnACID-E). In the third subsection, we modify the background modeling assumptions to introduce a convolutional structure and describe how to utilize this to derive an alternative fast online algorithm. Finally, we describe how motion correction, which is typically done as preprocessing step, can be performed online, and stream processing allows us to employ a very simple yet effective motion correction scheme. Throughout we use the common convention to denote vectors and matrices with boldface lowercase and uppercase letters respectively. We use *i*, *j* for general indexing. Where it helps the exposition, we use a different lowercase letter as index and the corresponding uppercase letter as its upper bound, e.g. *t* ∈ {1, …, *T*} as time index where *T* is the total number of frames observed, and *n* ∈ {1, …, *N*} as neuron index where *N* is the total number of neurons.

### CNMF for microendoscopic data (CNMF-E)

The recorded video data can be represented by a matrix $Y\in R_{+}^{d\times T}$, where *d* is the number of imaged pixels and *T* is the number of frames observed. Following [5], we model ***Y*** as

$$\begin{array}{c} Y=AC+B+E, \end{array}$$ (1)

where $A\in R_{+}^{d\times N}$ is a spatial matrix that encodes the location and shape of each neuron (spatial footprint), $C\in R_{+}^{N\times T}$ is a temporal matrix that characterizes the fluorescence of each neuron over time, matrix ***B*** represents background fluctuations and ***E*** is additive Gaussian noise with mean zero and diagonal covariance.

The CNMF framework of [5] incorporates further constraints beyond non-negativity. Each spatial footprint ***a***_*n*_ is constrained to be spatially localized and hence sparse. Similarly, the temporal components ***c***_*n*_ are highly structured, as they represent the cells’ fluorescence responses to typically sparse, nonnegative trains of action potentials. Following [23, 24], we model the calcium dynamics of each neuron ***c***_*n*_ with a stable autoregressive process of order *P*,

$$\begin{array}{c} c_{n}\left(t\right)=\sum_{p=1}^{P}\gamma_{p}c_{n}\left(t-p\right)+s_{n}\left(t\right), \end{array}$$ (2)

where *s*_*n*_(*t*)≥0 is the number of spikes that neuron *n* fired at the *t*-th frame, and *γ*_*p*_, *p* = 1, …, *P* correspond to the discrete time constants of the dynamics that depend on the kinematic properties of the used indicator.

For the case of microendoscopic data we refer the reader to [6] for a very detailed exposition of the model. The background ***B*** is modeled as sum of constant baselines ***B***^*c*^ and fluctuating activity ***B***^*f*^ [6]

$$\begin{array}{c} B=\underset{B^{c}}{\underset{︸}{\bar{b}1_{T}^{⊤}}}+\underset{B^{f}}{\underset{︸}{W(Y-AC-\bar{b}1_{T}^{⊤})}}, \end{array}$$ (3)

where **1**_*T*_ denotes a vector of *T* ones and the constant baselines are $\bar{b}=\frac{1}{T}\left(Y-AC\right)1_{T}$. The model for ***B***^*f*^ exploits that background sources (largely due to blurred out-of-focus fluorescence) are empirically much coarser spatially than the average neuron soma size. Thus we model ***B***^*f*^ at one pixel as a linear combination of the background fluorescence in pixels which are chosen to be nearby but not nearest neighbors, ***B***^*f*^ ≈ ***WB***^*f*^. ***W*** is an appropriate sparse weight matrix, where *W*_*ij*_ is constrained to *W*_*ij*_ = 0 if dist(***x***_*i*_, ***x***_*j*_) ∉ [*l*, *l* + 1[, thus we model the background at one pixel as a linear combination of the background fluorescence in pixels which are chosen to be on a ring with radius *l* [6]. Typically, *l* is chosen to be ∼1.5× the radius of an average neuron, to exclude contributions that might be affected from the activity of an underlying neuron.

### Fitting the CNMF-E model

We first recap the offline approach for fitting the CNMF-E model [6], and then show how it can be adapted to an online setup.

#### Offline

The estimation of all model variables can be formulated as a single optimization problem

$$\begin{array}{c} \underset{A,C,B}{\text{minimize}}\;∥Y-AC-B{∥}_{F}^{2}\;\text{subject}\;\text{to}\;\text{constraints.} \end{array}$$ (4)

The CNMF-E algorithm of [6] divides the nonconvex problem (4) into three simpler subproblems that are solved iteratively: Estimating ***A*** given estimates $\hat{C}$ and $\hat{B}$, estimating ***C*** given $\hat{A}$ and $\hat{B}$, and estimating ***B*** given $\hat{A}$ and $\hat{C}$.

***A*** and ***C*** are estimated using a modified version of “fast hierarchical alternating least squares” [25] that includes sparsity and localization constraints [26]. The update of ***A*** consists of block-coordinate decent steps iterating over neurons *n*,

$$\begin{array}{c} A_{p(n),n}\leftarrow {\left\lfloor A_{p(n),n}+\frac{{\left(\left(Y-\hat{B}\right){\hat{C}}^{⊤}\right)}_{p(n),n}-{\left(A\hat{C}{\hat{C}}^{⊤}\right)}_{p(n),n}}{{\left(\hat{C}{\hat{C}}^{⊤}\right)}_{nn}}\right\rfloor }_{+}, \end{array}$$ (5)

where ***p***(*n*) specifies the pixel indices where ***A***_:, *n*_ can take non-zero values, i.e. where neuron *n* is located. For computational efficiency the sufficient statistics $L=\left(Y-\hat{B}\right){\hat{C}}^{⊤}$ and $M=\hat{C}{\hat{C}}^{⊤}$ are computed only once initially and cached to be reused when iterating few times over neurons *n* ∈ {1, …, *N*}.

Similarly, the block-coordinate decent steps for updating ***C*** are

$$\begin{array}{c} C_{n,:}\leftarrow C_{n,:}+\frac{{\left({\hat{A}}^{⊤}\left(Y-\hat{B}\right)\right)}_{n,:}-{\left({\hat{A}}^{⊤}\hat{A}C\right)}_{n,:}}{{\left({\hat{A}}^{⊤}\hat{A}\right)}_{nn}}, \end{array}$$ (6)

with sufficient statistics ${\hat{A}}^{⊤}\left(Y-\hat{B}\right)$ and ${\hat{A}}^{⊤}\hat{A}$ computed only once initially. ***C*** should not merely be constrained to non-negative values but follow the dynamics of the calcium indicator, thus to further denoise and deconvolve the neural activity from the dynamics of the indicator the OASIS algorithm [23] is used. OASIS solves a modified LASSO problem

$$\begin{array}{c} \underset{c,s}{\text{minimize}}\;\frac{1}{2}{∥c-y∥}^{2}+\lambda {∥s∥}_{1}\;\text{subject}\;\text{to}\;s_{t}=c_{t}-\sum_{p=1}^{P}\gamma_{p}c_{t-p}\geq s_{\text{min}}\;\text{or}\;s_{t}=0, \end{array}$$ (7)

where ***y*** denotes a noisy neural calcium trace obtained as result of Eq (6). The *ℓ*_1_ penalty on ***s*** or the minimal spike size *s*_min_ can be used to enforce sparsity of the neural activity.

The spatiotemporal background is estimated from the linear regression problem

$$\begin{array}{c} {\underset{W}{\text{minimize}}\;∥X-WX∥}_{F}^{2}\;\;\text{subject}\;\text{to}\;W_{ij}=0\;\text{if}\;\text{dist}\left(x_{i},x_{j}\right)\notin [l,l+1[, \end{array}$$ (8)

where $X=Y-\hat{A}\hat{C}-\bar{b}1_{T}^{⊤}$ and $\bar{b}=\frac{1}{T}\left(Y-\hat{A}\hat{C}\right)1_{T}$. The solution is given by the normal equations for each pixel *i*,

$$\begin{array}{c} W_{i,r_{l}\left(i\right)}={\left(XX^{⊤}\right)}_{i,r_{l}\left(i\right)}{\left(XX^{⊤}\right)}_{r_{l}\left(i\right),r_{l}\left(i\right)}^{-1}, \end{array}$$ (9)

where ***r***_*l*_(*i*) = {*j*|dist(***x***_*i*_, ***x***_*j*_)∈[*l*, *l* + 1[} specifies the pixel indices where ***W***_*i*,:_ can take non-zero values. Given the optimized ***W***, the whole background signal is $B=WX+\bar{b}1_{T}^{⊤}$. More information can be found in [6].

#### Online

The offline framework presented above can be adapted to a data streaming setup, using the same model assumptions. Instead of running CNMF-E afresh on the entire data seen so far up to time *t*, ***Y***[:, 1: *t*], the previous estimates ***A***, ***C***, ***B*** obtained on the data up to time *t* − 1, ***Y***[:, 1: *t* − 1], are updated using the newly recorded frame ***y***_*t*_, eliminating the need to load the entire data ***Y*** in memory and avoiding repetitive computations. One complicating factor is that during online processing some neurons may become active for the first time, thus we need a method to detect those new components and append them to the spatial and temporal matrices ***A*** and ***C***. In essence, we need to appropriately modify the online algorithm for analyzing 2-photon calcium imaging data (OnACID, [21]) to the case of microendoscopic 1-photon data which requires a more refined background model.

Using Eq (1), the observed fluorescence at time *t* can be written as

$$\begin{array}{c} y_{t}=Ac_{t}+b_{t}+\varepsilon_{t}. \end{array}$$ (10)

The (non-deconvolved) activity of all neurons at time *t*, ***c***_*t*_, is obtained by iteratively evaluating Eq (6) given raw frame data ***y***_*t*_, spatial footprints ***A***, and background parameters $W,\bar{b}$. The activity is further denoised and deconvolved by running OASIS [23], which is not only a very fast algorithm, but crucially progresses through each time series sequentially from beginning to end and is thus directly applicable to stream processing. Using the expression of ***b***_*t*_ (the *t*-th column of ***B***) from Eq (3), the background term in Eq (6) evaluates to $A^{⊤}b_{t}=A^{⊤}Wy_{t}-A^{⊤}WAc_{t}-A^{⊤}W\bar{b}+A^{⊤}\bar{b}$ and for computational efficiency the terms ***A***^⊤^ ***W***, ***A***^⊤^***WA*** and $A^{⊤}\left(W\bar{b}-\bar{b}\right)$ are maintained in memory and updated incrementally, cf. S1 Appendix. Warm starts are exploited by initializing ***c***_*t*_ with the value at the previous frame ***c***_*t*−1_, since the calcium traces ***C*** are continuous and typically change slowly. Moreover, the temporal traces of components that do not spatially overlap with each other can be updated simultaneously in vector form; we use a simple greedy scheme to partition the components into spatially non-overlapping groups [21].

The spatial footprints ***A*** are obtained by iteratively evaluating Eq (5) and can be estimated efficiently as in [22] by only keeping in memory the sufficient statistics

$$\begin{array}{c} L_{t}=\frac{t-1}{t}L_{t-1}+\frac{1}{t}\left(y_{t}-b_{t}\right)c_{t}^{⊤},\;M_{t}=\frac{t-1}{t}M_{t-1}+\frac{1}{t}c_{t}c_{t}^{⊤}. \end{array}$$ (11)

Since neurons’ shapes are not expected to change at a fast timescale, updating ***A*** is actually not required at every timepoint; in practice we update every 200 time steps, again warm started at the value from the previous iteration, cf. Alg 1. Additionally, the sufficient statistics ***L***_*t*_, ***M***_*t*_ are only needed for updating the estimates of ***A*** so they can be updated only when required (using computationally efficient matrix products). Further, only a sparse subset of the elements of matrix ***L*** needs to be updated, because Eq (5) does not access all elements of ***L***, but only elements ***p***(*n*) for each column *n* of ***L*** (i.e. only pixel indices ***p***(*n*) where neuron *n* is located). Hence, we speed up the algorithm by updating only those few entries of ***L***, cf. S1 Appendix.

To update the background components $W,\bar{b}$, we keep track of the constant baselines $\bar{b}$ and the sufficient statistics ***χ*** = ***XX***^⊤^ that is needed to compute ***W*** using Eq (9)

$$\begin{array}{c} {\bar{b}}_{t}\leftarrow \frac{t-1}{t}{\bar{b}}_{t-1}+\frac{1}{t}\left(y_{t}-Ac_{t}\right),\;\chi_{t}=\frac{t-1}{t}\chi_{t-1}+\frac{1}{t}x_{t}x_{t}^{⊤}, \end{array}$$ (12)

where $x_{t}=y_{t}-Ac_{t}-{\bar{b}}_{t}$. As is the case with the spatial footprints, updating the background is actually not required at every timepoint and in practice we update every 200 time steps, cf. Alg 1 and S1 Appendix. Processing pixel *i* according to Eq (9) (see also S1 Appendix) accesses only vector $\chi_{i,r_{l}(i)}$ and sub-matrix $\chi_{r_{l}(i),r_{l}(i)}$. Some elements of ***χ*** are not part of any sub-matrix or vector for any *i* and thus are never accessed. In practice we therefore update and store only these vectors and sub-matrices for computational and memory efficiency. Because the background has no high spatial frequency components, it can be spatially decimated to further speed up processing [23] without compromising the quality of the results. E.g. downscaling by a factor of 2 reduces the number of pixels by a factor of 4 and the number of elements in ***W*** and ***χ*** by a factor of 16. Less and smaller least squares problems (Eq 9) need to be solved, which drastically reduces processing time and memory consumption.

Note that updating the background components and all the spatial footprints at a given frame results in a computational bottleneck for that specific frame. While on average, this effect is minimal (cf. Results section) a temporary slowdown can have an adverse effect on a real-time closed loop setup. This restriction can be lifted by holding the background model fixed and updating the spatial footprints in a distributed manner across all frames. As described later, using a lower dimensional background model can achieve that and enable fast real time processing with balanced workload across all frames.

To initialize our algorithm we use the CNMF-E algorithm on a short initial batch of data of length *T*_*b*_, (e.g., *T*_*b*_ = 200). The sufficient statistics are initialized from the components that the offline algorithm finds according to Eqs (11) and (12).

**Algorithm 1** OnACID-E

**Require**: Data matrix ***Y***, initial estimates $A,C,S,W,\bar{b}$, current number of components *N*, current time step *t*′, rest of parameters.

1: $X=Y\left[:,1:t^{\prime }\right]-AC-\bar{b}1_{t^{\prime }}^{⊤}$

2: ***R***_buf_ = (***X*** − ***WX***)[:, *t*′ − *l*_*b*_ + 1: *t*′]   ⊳ Initialize residual buffer

3: ***χ*** = ***XX***^⊤^           ⊳ Initialize sufficient statistics

4: ***L*** = ***Y***[:, 1: *t*′]***C***^⊤^/*t*′

5: ***M*** = ***CC***^⊤^/*t*′

6: G=DetermineGroups(A,N)    ⊳ [21]

7: *t* = *t*′

8: **while** there is more data **do**

9:  *t* ← *t* + 1

10:  ***y***_*t*_ ← AlignFrame(***y***_*t*_, ***Ac***_*t*−1_)    ⊳ Alg S6

11:  $c_{t}\leftarrow \text{UpdateTraces}\left(A,c_{t-1},y_{t},W,\bar{b},G\right)$    ⊳ Alg S1

12:  ***C***, ***S*** ← OASIS(***C***, ***γ***, ***s***_min_, *λ*)    ⊳ [23]

13:  $\bar{b}\leftarrow \frac{t-1}{t}\bar{b}+\frac{1}{t}\left(y_{t}-Ac_{t}\right)$

14:  $A,C,N,G,R_{\text{buf}}\leftarrow \text{DetectNewComponents}(A,C,W,\bar{b},N,G,R_{\text{buf}},y_{t})$   ⊳ Alg S5

15:  **if** $\;\mathrm{mod}\;(t-t^{\prime },T_{p})=0$ **then**    ⊳ Update ***χ***, ***L***, ***M***, ***W***, ***A*** every *T*_*p*_ time steps

16:   $\chi ,L,M\leftarrow \text{UpdateSuffStatistics}(Y\left[:,t-T_{p}+1:t\right],C\left[:,t-T_{p}+1:t\right],W,\bar{b},A,\chi ,L,M)$    ⊳ Alg S2

17:   ***W*** ← UpdateBackground(***χ***)    ⊳ Alg S4

18:   ***A*** ← UpdateShapes(***L***, ***M***, ***A***)    ⊳ Alg S3

19: **return** $A,C,S,W,\bar{b}$

#### Detecting new components

The approach explained above enables tracking the activity of a fixed number of sources, and will ignore neurons that become active later in the experiment. Following [21], we approach the problem by introducing a buffer ***R***_buf_ that contains the last *l*_*b*_ instances of the residual signal ***r***_*t*_ = ***y***_*t*_ − ***Ac***_*t*_ − ***b***_*t*_ $\left(R_{\text{buf}}=[r_{t-l_{b}+1},\ldots r_{t}]\right)$, where *l*_*b*_ is a reasonably small number, e.g., *l*_*b*_ = 100. From this buffer we compute a summary image (as detailed later we actually update the summary image instead of computing it afresh) and then search for the local maxima of the image to determine new candidate neurons.

One option for the summary image ***e*** is to proceed along the lines of [5], i.e. to perform spatial smoothing with a Gaussian kernel with radius similar to the expected neuron radius, and then calculate the energy for each pixel *i*, $e\left[i\right]=\frac{1}{l_{b}}\sum_{t}\mathrm{filt}{\left(R_{\text{buf}}\left[i,t\right]\right)}^{2}$, where filt() refers to the smoothing operation. Another option is to follow [6] and calculate the peak-to-noise ratio (PNR),

$$\begin{array}{c} i_{\text{pnr}}\left[i\right]=\frac{\max_{t}R_{\text{buf}}\left[i,t\right]}{\sigma_{i}}, \end{array}$$ (13)

as well as the local cross-correlation image,

$$\begin{array}{c} i_{\text{corr}}\left[i\right]=\frac{1}{\left|N(i)\right|}\sum_{j\in N(i)}\text{corr}\left(R_{\text{buf}}\left[i,:\right],R_{\text{buf}}\left[j,:\right]\right), \end{array}$$ (14)

where N(i) specifies the neighboring pixels of pixel *i* and the function corr() refers to Pearson correlation. Their pixel-wise product ***e*** = ***i***_pnr_ ⊙ ***i***_corr_ is used as summary image. We use the latter throughout the Results section, if not explicitly stated otherwise. New candidate components ***a***_new_, and ***c***_new_ are estimated by performing a local rank-1 NMF of the residual matrix restricted to a fixed neighborhood around the point of maximal variance, or maximal product of PNR and cross-correlation, respectively.

To limit false positives, the candidate component is screened for quality. Similarly to [21], to prevent noise overfitting, the shape ***a***_new_ must be significantly correlated (e.g., *θ*_sp_ ∼ 0.5) to the residual buffer averaged over time and restricted to the spatial extent of ***a***_new_. Moreover, if ***a***_new_ significantly overlaps with any of the existing components, then its temporal component ***c***_new_ must not be highly correlated with the corresponding temporal components; otherwise we reject it as a possible duplicate of an existing component. Further, for a candidate component to correspond to an active neuron its trace must exhibit dynamics reminiscent of the calcium indicator’s transient. As criterion for this we require the SNR of trace ***c***_new_ to be above a certain threshold *θ*_SNR_. We tuned the spatial and temporal thresholds for each considered dataset. Once a new component is accepted, ***A***, ***C*** are augmented with ***a***_new_ and ***c***_new_ respectively, the quantities ***A***^⊤^ ***W***, ***A***^⊤^ ***WA*** and $A^{⊤}\left(W\bar{b}-\bar{b}\right)$ are updated via augmentation, and the sufficient statistics are updated as follows:

$$\begin{array}{c} L_{t}=[L_{t},\frac{1}{t}\left(Y_{\text{buf}}-B_{\text{buf}}\right)c_{\text{new}}^{⊤}],\;M_{t}=\frac{1}{t}[\begin{array}{cc} tM_{t} & C_{\text{buf}}c_{\text{new}}^{⊤}\\ c_{\text{new}}C_{\text{buf}}^{⊤} & ∥c_{\text{new}}{∥}^{2} \end{array}], \end{array}$$ (15)

where $Y_{\text{buf}},C_{\text{buf}},B_{\text{buf}}=\bar{b}1_{l_{b}}^{⊤}+W\left(Y_{\text{buf}}-AC_{\text{buf}}-\bar{b}1_{l_{b}}^{⊤}\right)$ denote the matrices ***Y***, ***C***, ***B***, restricted to the last *l*_*b*_ frames that the buffer stores. This process is repeated until all candidates have been screened, at which point the next frame is read and processed. The process is summarized in Alg S5.

#### Updating the summary image

For computational efficiency we avoid repeated computations and perform incremental updates of the summary image instead of computing it afresh. If the variance image is used, it is updated according to $e\leftarrow e+\frac{1}{l_{b}}\left(\mathrm{filt}{\left(r_{t}\right)}^{2}-\mathrm{filt}{\left(r_{t-l_{b}}\right)}^{2}\right)$ when the next frame is processed. When a new component with footprint ***a*** is added the residual changes at the component’s location and we update the variance image accordingly locally only for pixels *i* where the smoothed component is positive (filt(***a***)[*i*] > 0) according to $e\left[i\right]\leftarrow \frac{1}{l_{b}}\sum_{t}\mathrm{filt}{\left(R_{\text{buf}}\left[i,t\right]\right)}^{2}$.

Next we consider the case that the product of cross-correlation image and PNR image is used as summary image. We keep track of the first and second order statistics

$$\begin{array}{c} \mu_{i}=\frac{1}{l_{b}}\sum_{t}R_{\text{buf}}\left[i,t\right]\;\text{and}\;\nu_{ij}=\frac{1}{l_{b}}\sum_{t}R_{\text{buf}}\left[i,t\right]R_{\text{buf}}\left[j,t\right], \end{array}$$ (16)

the latter only for pixels j∈{i}∪N(i). These statistics are updated according to

$$\begin{array}{cc} \mu & \leftarrow \mu +\frac{1}{l_{b}}\left(r_{t}-r_{t-l_{b}}\right) \end{array}$$ (17)

$$\begin{array}{cc} \nu_{ij} & \leftarrow \nu_{ij}+\frac{1}{l_{b}}{\left(r_{t}r_{t}^{⊤}-r_{t-l_{b}}r_{t-l_{b}}^{⊤}\right)}_{ij} \end{array}$$ (18)

when the next frame is processed. The cross-correlation values are computed from these statistics as

$$\begin{array}{c} \text{corr}\left(R_{\text{buf}}\left[i,:\right],R_{\text{buf}}\left[j,:\right]\right)=\frac{\nu_{ij}-\mu_{i}\mu_{j}}{\sqrt{\left(\nu_{ii}-\mu_{i}^{2}\right)\left(\nu_{jj}-\mu_{j}^{2}\right)}}, \end{array}$$ (19)

and the correlation image is obtained according to Eq (14). For computing the PNR image we use the noise level *σ*_*i*_ estimated on the small initial batch for the denominator in Eq (13) and keep track of the maximum image ***i***_max_ ← max(***i***_max_, ***r***_*t*_) for the nominator. When a new component with footprint ***a*** and time series $\tilde{c}$ is added we set ***i***_max_[*i*] to zeros if *a*_*i*_ > 0. The statistics for the cross-correlation are updated as

$$\begin{array}{cc} \mu_{i} & \leftarrow \mu_{i}-\frac{1}{l_{b}}\sum_{t}{\tilde{c}}_{t}a_{i} \end{array}$$ (20)

$$\begin{array}{cc} \nu_{ij} & \leftarrow \nu_{ij}+\frac{1}{l_{b}}\sum_{t}\left({\tilde{c}}_{t}^{2}a_{i}a_{j}-R_{\text{buf}}\left[j,t\right]{\tilde{c}}_{t}a_{i}-R_{\text{buf}}\left[i,t\right]{\tilde{c}}_{t}a_{j}\right). \end{array}$$ (21)

The whole online procedure of OnACID-E is described in Algorithm 1; S1 Appendix includes pseudocode description of the referenced routines.

### Background modeling using convolutional neural networks

The background model used in the CNMF-E algorithm (Eq 8) assumes that the value of the background signal at a given point in space is given by a linear combination of the background values from the points in a ring centered around that pixel with width 1 and radius *l*, where *l* is larger than the radius of the typical neuron in the dataset by a small factor (e.g. 1.5) plus a pixel dependent scalar [6]. While powerful in practice, this model does not assume any dependence between the linear combination weights of all the different pixels, and results in a model with a very large number of parameters to be estimated. Ignoring pixels near the boundary, each row of the matrix ***W*** which represents the linear combination weights will have approximately [2*πl*] non-zero entries (where [⋅] denotes the integer part), giving a total number of *d*([2*πl*] + 1) parameters to be estimated. While this estimation can be done efficiently in parallel as discussed above, and the overall number of parameters can be reduced through spatial downsampling, we expect that the overall number of degrees of freedom in such a model is much lower. The reason is that the ring model of CNMF-E aims to capture aspects of the point spread function (PSF) which is largely invariant with respect to the location within the local environment of each neuron.

To test this hypothesis we used a very simple convolutional neural network (CNN) with ring shaped kernels to capture the background structure. The intuition behind the convolution is straightforward: if all the rows of the ***W*** matrix had the same non-zero entries (but centered around different points) then the application of ***W*** would correspond to a simple spatial convolution with the common “ring” as the filter. In our case this model is not expressive enough to adequately fit the background, in particular it fails to capture pixel dependent brightness differences, and by assuming shift invariance it fails to capture that the PSF can vary when compared across the full FOV. Therefore we investigated parametrizing the background model with a slightly more complex model, which we refer to as “Ring-CNN”.

Let $f_{\theta }:R^{d}\mapsto R^{d}$ be a function that models the autoregressive nature of the background. In the CNMF-E case this simply corresponds to $f_{\theta }\left(y-Ac\right)=W\left(y-Ac-\bar{b}\right)+\bar{b}$, cf. Eq (3). In the linear model we parametrize the function as

$$\begin{array}{c} f_{\theta }\left(y\right)=\sum_{k=1}^{K}w_{k}⊙\left(h_{k}*y\right)+\bar{b}, \end{array}$$ (22)

where $\bar{b},w_{k}\in R^{d},k=1,\ldots ,K$, and ⊙, * refer to pointwise multiplication and spatial convolution, respectively (with slight abuse of notation we assume that ***y*** has been reshaped back to 2d image to perform the convolution and the result of the convolution is again vectorized). Finally, ***h***_*k*_, *k* = 1, …, *K* is a ring shaped convolutional kernel which takes non-zero values only at a specified annulus around its center. Note that this corresponds to parametrizing directly ***W*** as

$$\begin{array}{c} W=\sum_{k=1}^{K}w_{k}⊙H_{k}, \end{array}$$ (23)

where $H_{k}\in R^{d\times d}$ is the matrix induced by the convolutional kernel ***h***_*k*_. Constructing the sparse matrix ***W*** explicitly (and efficiently using diagonal storage) can speed up evaluation of the background when a GPU is not available. Intuitively this model corresponds to using a pixel dependent linear combination of *K* ring basis functions, and results in a total *K*(*d* + [2*πl*]) + *d* parameters to be estimated. Compared to the *d*([2*πl*] + 1) number of parameters for the CNMF-E model, this can result in a significant reduction when *K* < [2*πl*].

Note that decoupling the number of different “rings” from the total number of pixels, enables the consideration of wider “rings” that integrate over a larger area of the FOV and can potentially provide more accurate estimates, without a dramatic increase on the number of parameters to be learned. For example, a “ring” with inner radius *l* and width *w* would require approximately [*πw*(2*l* + *w* − 1)] parameters and the total number of parameters would be *K*([*πw*(2*l* + *w* − 1)] + *d*) as opposed to *d*([*πw*(2*l* + *w* − 1)] + 1) for the standard CNMF-E model.

#### Unsupervised training on the raw data

To estimate the autoregressive background model in the CNMF-E algorithm, we want to operate on the data after the spatiotemporal activity of all detected neurons has been removed (Eq 8). For the CNN model this would translate into the optimization problem

$$\begin{array}{c} \hat{\theta }=\underset{\theta }{arg min}\;L\left(Y-AC,f_{\theta }\left(Y-AC\right)\right), \end{array}$$ (24)

where $L\left(\cdot ,\cdot \right):R^{d\times T}\times R^{d\times T}\mapsto R_{+}$ is an appropriate loss function (e.g. the Frobenius norm).

For the CNMF-E algorithm, operating on ***Y*** − ***AC*** is necessary because each “ring” has its own independent weights whose estimation can be biased from the activity of nearby neurons. In the CNN case however, the background model assumes a significant amount of weight sharing between the different “rings” which makes the estimation more robust to the underlying neural activity. Therefore we can estimate the background model by solving directly

$$\begin{array}{c} \hat{\theta }=\underset{\theta }{arg min}\;L\left(Y,f_{\theta }\left(Y\right)\right), \end{array}$$ (25)

for an appropriately choosen loss function L meaning that the solution of Eq (25) should satisfy $f_{\hat{\theta }}\left(Y\right)\approx Y-AC$. Because $Y-AC\approx f_{\hat{\theta }}\left(Y-AC\right)=f_{\hat{\theta }}\left(Y\right)-f_{\hat{\theta }}\left(AC\right)$, the underlying assumption is that $f_{\hat{\theta }}\left(AC\right)$ can be neglected. While the autoregressive model can capture the background well, i.e. $Y-AC\approx f_{\hat{\theta }}\left(Y-AC\right)$, this is not the case for the neural activity, because the fluorescence ***AC*** at each pixel due to neural activity can not be reconstructed well using the unrelated fluorescence traces on the ring around that pixel $f_{\hat{\theta }}\left(AC\right)$, i.e. $AC≉f_{\hat{\theta }}\left(AC\right)$ independent of $\hat{\theta }$. This particularly holds during training of the CNN where not just one but hundreds of frames are reconstructed, ruling out overfitting. Thus including the neural activity ***AC*** in the objective, Eq (25) instead of Eq (24), hardly affects the optimal parameters $\hat{\theta }$. Furthermore, since ***AC*** is nonnegative we seek to under-approximate ***Y*** with the background *f*_***θ***_(***Y***). To encode that in the objective function we can consider a quantile loss function [27] that penalizes over-approximation more than under-approximation:

$$\begin{array}{c} l_{q}\left(x,y\right)=\{\begin{array}{cc} q(x-y), & x\geq y\\ (1-q)(y-x), & x<y \end{array}, \end{array}$$ (26)

For some *q* ∈ (0, 1] and take $L\left(X,Y\right)=2\sum_{i,j}l_{q}\left(X_{ij},Y_{ij}\right)$. For example, for *q* = 0.5 Eq (26) corresponds to the *L*_1_ norm of the difference, and to promote the under-approximation property we use *q* < 0.5. Since the models are differentiable and the objective function is additive, Eq (25) can be optimized in an online mode using stochastic gradient descent.

#### Online processing

**Algorithm 2** Online processing with a Ring-CNN background model

**Require**: Data matrix ***Y***, number of initial timesteps *T*_init_, rest of parameters.

1: ***X*** = MotionCorrect(***Y***[:, 1 : *T*_init_])            ⊳ [28]

2: $\hat{\theta }\leftarrow {arg min}_{\theta }L\left(X,f_{\theta }\left(X\right)\right)$   ⊳ Estimate ring CNN (25)

3: $X\leftarrow X-f_{\hat{\theta }}\left(X\right)$            ⊳ Filter Background

4: ***A***, ***C***, ***S***, **b**, **f**, = InitializeOnline2P(***X***)   ⊳ Initialize online algorithm [17]

5: *t* = *T*_init_

6: **while** there is more data **do**

7:  *t* ← *t* + 1

8:  ***y***_*t*_ ← AlignFrame(***y***_*t*_, ***b***_*t*−1_ + ***Ac***_*t*−1_)           ⊳ Alg S6

9:  $x_{t}=y_{t}-f_{\hat{\theta }}\left(y_{t}-Ac_{t-1}\right)$   ⊳ Remove background from current frame

10:  [***c***_*t*_; **f**_*t*_] ← UpdateTraces2P(***A***, [***c***_*t*−1_; **f**_*t*−1_], ***x***_*t*_, ***b***, **f**)   ⊳ [21, Alg S3]

11:  ***C***, ***S*** ← OASIS(***C***, *γ*, ***s***_min_, λ)             ⊳ [23]

12:  ***A***, ***C***, *N*, ***R***_buf_← DetectNewComponents2P(***A***, ***C***, ***R***_buf_, ***x***_*t*_)  ⊳ [21, Alg S4]

13:  [***A***, ***b***] ← UpdateShapes2P(***L***, ***M***, [***A***, ***b***])         ⊳ [21, Alg S5]

14:  **if** mod (*t* − *T*_init_, *T*_*p*_) = 0 **then**      ⊳ Update ***L***, ***M*** every *T*_*p*_ time steps

15:   ***L***, ***M*** ← UpdateSuffStatistics2P(***Y***, ***C***, ***A***, ***L***, ***M***)       ⊳ [21]

16: **return** $A,C,S,b,f,\hat{\theta }$

In practice, we found that by using rings of increased width (e.g. 5 pixels), training the model only during the initialization process on a small batch frames, leads to convergence due to the large amount of weight sharing that reduces the number of parameters. Once the model has been trained, it can be used to remove the background from the data (after motion correction). To reduce the effect of active neurons on the inferred background we can approximate the activity at time *t*, with the activity at time *t* − 1, and subtract that from the data frame prior to computing the background. In other words, we can use the approximation

$$\begin{array}{c} b_{t}≃f_{\hat{\theta }}\left(y_{t}-Ac_{t-1}\right). \end{array}$$ (27)

Once the background has been removed, online processing can be done using the standard online algorithm for two-photon data [21]. The process is summarized in Alg 2, where the suffix “2P” has been added to some routines to indicate their differences compared to the routines used in OnACID-E that are slightly more complicated due to their additional background treatment step. Note that although the focus of this paper is on online processing, the ring-CNN background model can also be used to derive an offline algorithm for microendoscopic 1p data.

### Online motion correction

Similarly to [21], online motion correction can be achieved by using the previously denoised frame ***b***_*t*−1_ + ***Ac***_*t*−1_ to derive a template for registering ***y***_*t*_. In practice, we observed that this registration process is more robust to drift introduced by corrupt frames when an average of the past *M* denoised frames is used as a template, with *M* ∼ 50. As proposed in [17], passing both the template and the frame through a high pass spatial filter can suppress the strong background signal present in microendoscopic 1-photon data, and lead to more accurate computation of the alignment transformation. Rigid or piecewise rigid translations can be estimated as described in [28]. The inferred transformation is then applied to original frame ***y***_*t*_. The process is summarized in Alg S6.

### Analysis details

The detection of components in CNMF-E is controlled by thresholds on the minimum peak-to-noise ratio of seed pixels, *P*_min_, and on the minimum local correlation of seed pixels, *L*_min_. OnACID-E imposes a threshold ***θ***_sp_ on the correlation between a component’s spatial footprint and the data averaged over time, as well as a threshold ***θ***_SNR_ on the signal to noise ratio. These parameters should be adjusted for the considered dataset and are listed in Table 1. *P*_min_ and *L*_min_ can be adjusted based on inspection of the PNR and cross-correlation summary images over the initial batch to account for different noise levels between data sets.

**Table 1:** Thresholds controlling the detection of components for the analyzed datasets.

Dataset	Striatum	PFC	Hippocampus	BNST
*L*_min_	0.7	0.9	0.9	0.92
*P*_min_	7	15	15	15
*θ*_sp_	0.55	0.9	0.85	0.6
*θ*_SNR_	3.5	2.8	2.8	3.5

To compare the results between two algorithms we registered the components using the method implemented in CaImAn (with default parameters) and described in [17]. It constructs a matrix of pairwise distances between two components and computes an optimal matching between the components using the Hungarian algorithm to solve the linear assignment problem. Components detected by both algorithms are true positives (TP). Components additionally detected by the first algorithm are false negatives (FN), and false positives (FP) for the second. The accuracy of the agreement is measured by the F1-Score, which is defined as the harmonic mean of the precision and recall, and is in terms of type I and type II errors given by

$$\begin{array}{c} F_{1}=\frac{2\text{TP}}{2\text{TP}+\text{FP}+\text{FN}}. \end{array}$$ (28)

The output of the ring CNN is invariant to rotations of its parameters: Assembling the (vectorized) kernels into a matrix ***H*** ≔ [***h***_1_, …, ***h***_*K*_] we obtain new kernels $\tilde{H}$ by multiplying with a rotation matrix ***R***, $\tilde{H}=HR$. Likewise, assembling the weights into a matrix ***W*** ≔ [***w***_1_, …, ***w***_*K*_] we obtain new weights $\tilde{W}=WR$. The rotation leaves the matrix product $\tilde{W}{\tilde{H}}^{⊤}=WR{\left(HR\right)}^{⊤}=WRR^{⊤}H^{⊤}=WH^{⊤}$ invariant, and thus does not change the output of the CNN. To obtain convolution kernels that are ordered by ‘importance’ we can perform a singular value decomposition (SVD) of ***W*** ***H***^⊤^ = ***USV***^⊤^, where the singular values in the diagonal matrix **S** are ordered form largest to smallest. We set $\tilde{W}=US_{u}$, ${\tilde{H}}^{⊤}=S_{v}V^{⊤}$, with diagonal scaling matrices ***S***_u_, ***S***_v_ such that ***S*** = ***S***_u_ ***S***_v_ and depict those in the Results section. Note that the SVD approach would also allow to post-select the number of convolution kernels *K*. One could start with an upper estimate of *K*, train the CNN, perform SVD of ***W H***^⊤^, and only keep the singular vectors for which the singular value is above some threshold.

## Results

### Online analysis of 1p microendoscopic data using OnACID-E

We tested the online CNMF-E implementation of OnACID-E on in vivo microendosopic data from mouse dorsal striatum, with neurons expressing GCaMP6f. The data was acquired while the mouse was freely moving in an open field arena. The dataset consisted of 6000 frames at 10 Hz resolution (for further details refer to [6, 29]). We initialized the online algorithm by running CNMF-E on the first 200 frames.

**Fig 1:**
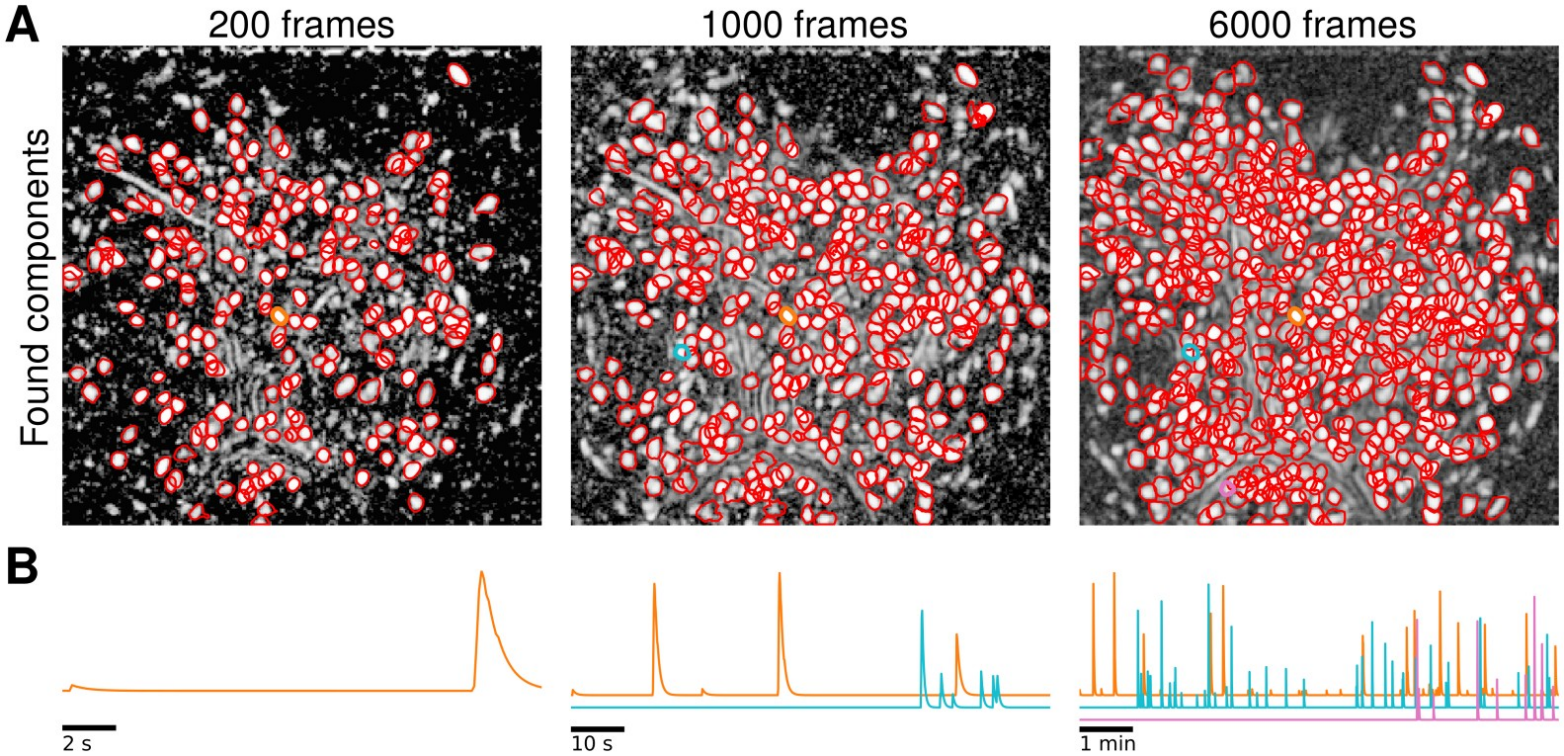
Illustration of the online data analysis process. Snapshots of the online analysis after processing 200 frames (left), 1000 frames (middle), and 6000 frames (right). **(A)** Contours of the components (neurons and processes) found by OnACID-E up to each snapshot point, overlaid over the local cross-correlation image of the spatially filtered data [6] at that point. **(B)** Examples of neuron activity traces (marked by corresponding colors in panel A). As the experiment proceeds, OnACID-E detects newly active neurons and tracks their activity. A video showing the whole online analysis can be found at S1 Video.

We illustrate OnACID-E in process in Fig 1. At the beginning of the experiment (Fig 1 left), only some components are active, as shown in panel A by the correlation image computed using the spatially filtered data [6], and most of these are detected by the algorithm (N = 218), while avoiding false positives. As the experiment proceeds more neurons activate and are subsequently detected by OnACID-E (Fig 1 middle, N = 337, and right, N = 554), which also tracks their activity across time (Fig 1B). See also S1 Video for further illustration.

### Comparison of OnACID-E with CNMF-E

**Fig 2:**
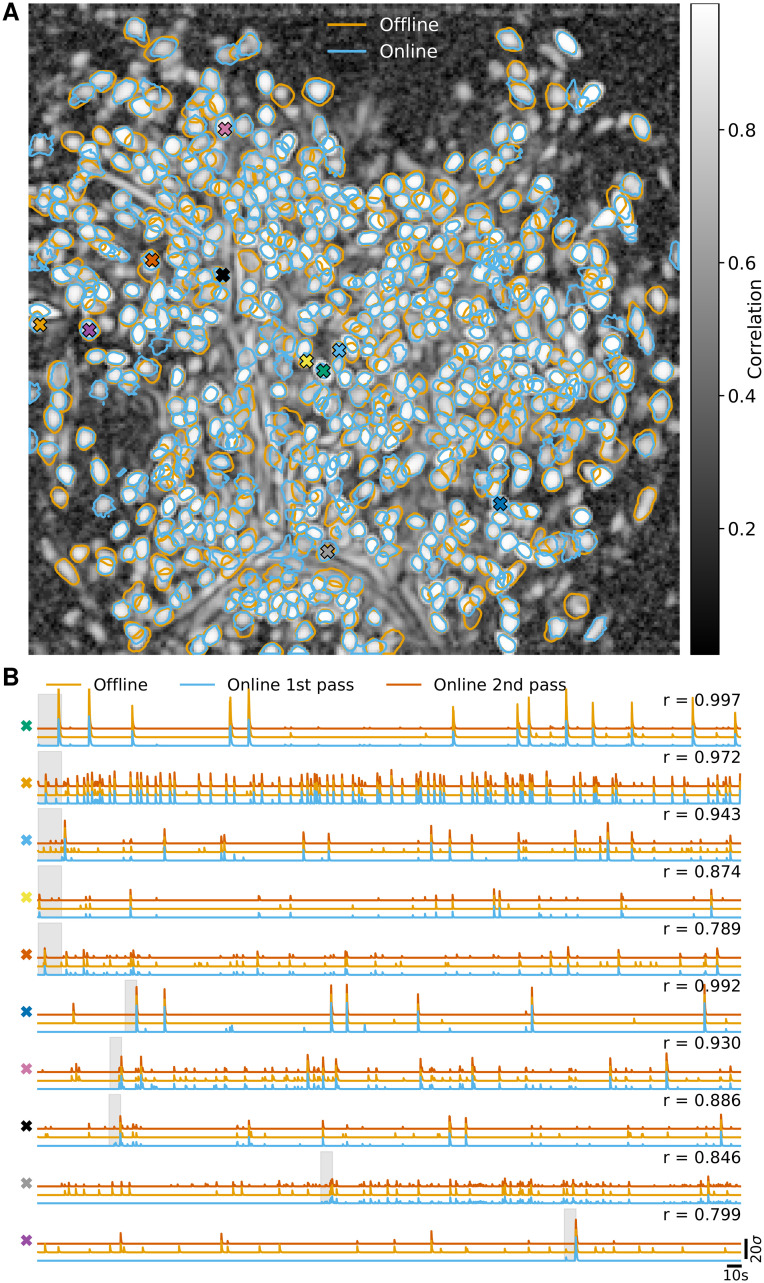
Comparison of OnACID-E with CNMF-E on data from neurons expressing GCaMP6f recorded *in vivo* in mouse dorsal striatum area. **(A)** Contour plots of all neurons detected by CNMF-E using patches (orange) and OnACID-E (blue), overlaid over the local cross-correlation image. Colors match the example traces shown in **(B)**, which illustrate the temporal components of ten example neurons detected by both implementations. The first five have been detected in the initialization phase, the last five during online processing. The gray shaded area shows the mini-batch OnACID-E used for the cell’s initialization, thus the area’s right border indicates at what frame the cell was initialized. The numbers to the upper right of each trace shows the correlation *r* between ‘offline’ and ‘online 2nd pass’.

In Fig 2 we report the results of the analysis using OnACID-E and compare to the results of CNMF-E with patches, i.e. the field of view (FOV) is split into smaller overlapping patches that are processed in parallel and combined at the end [17]. For each algorithm, after the processing was done, the identified components were merged, and then screened for false positive using the tests employed in the CaImAn package. Both implementations detect similar components (Fig 2A) with an F1-score of 0.891 (0.875 if the variance summary image was used to detect new components). 506 components were found in common by both implementations. 48 and 76 additional components were detected by OnACID-E and CNMF-E respectively. The additional components are depicted in S1 and S2 Figs respectively, and most of them appear to be actual cells. Ten example temporal traces are plotted in Fig 2B. The first five are from neurons that have been detected in the initialization phase, the last five during online processing. Not every neuron was detected immediately once it became active (blue traces); low activity events can be too weak to trigger detection as new component, but are accurately captured once the existence of the neuron has already been established.

Hence, when the data is analyzed after the experiment, e.g. when OnACID-E is used instead of CNMF-E for the sake of available computing resources (see below), one can perform a second online pass over the dataset, initialized with the results of the first pass, to recover the entire activity traces (red). The median correlation between the temporal traces of neurons detected by both implementations was 0.852 ± 0.008 (median ± standard error of the median).

**Fig 3:**
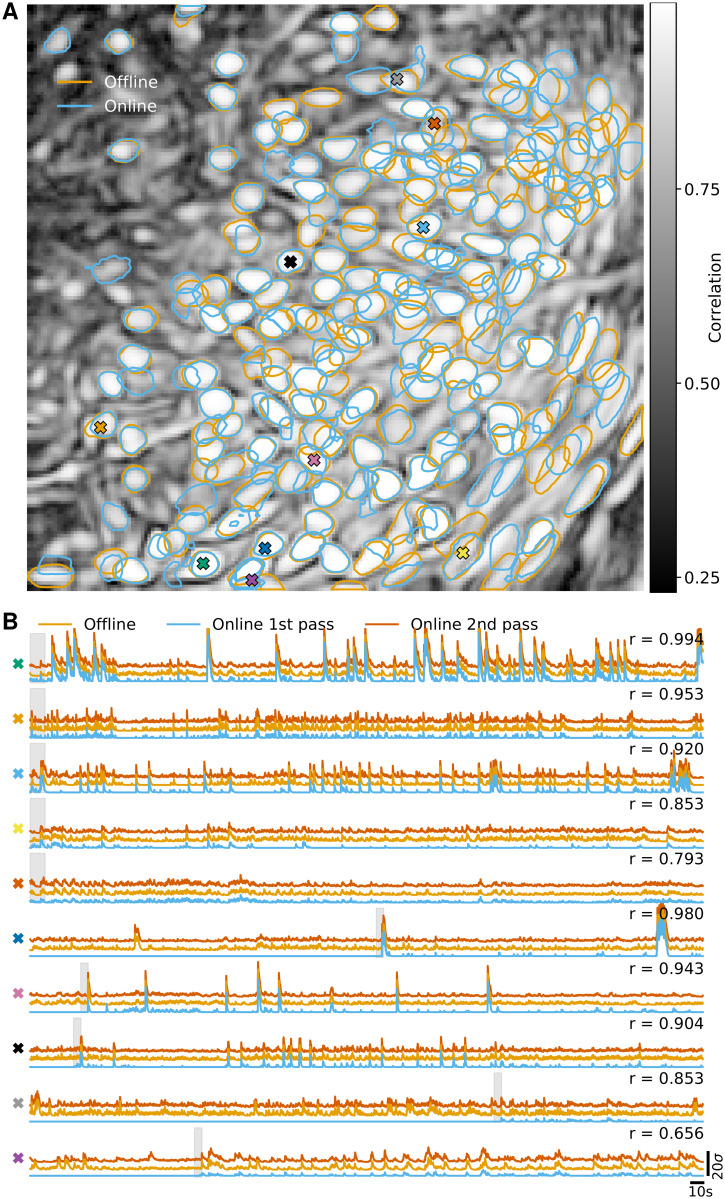
Comparison of OnACID-E with CNMF-E on data from neurons expressing GCaMP6s recorded *in vivo* in mouse prefrontal cortex. **(A)** Contour plots of all neurons detected by CNMF-E using patches (orange) and OnACID-E (blue), overlaid over the local cross-correlation image. Colors match the example traces shown in **(B)**, which illustrate the temporal components of ten example neurons detected by both implementations. The gray shaded area shows the mini-batch OnACID-E used for the cell’s initialization, thus the area’s right border indicates at what frame the cell was initialized. The numbers to the upper right of each trace shows the correlation *r* between ‘offline’ and ‘online 2nd pass’.

We repeated the analysis on *in vivo* microendosopic data from neurons expressing GCaMP6s in prefrontal cortex of a freely behaving mouse. This second dataset consisted of 9000 frames at 15 Hz resolution (for further details refer to [6]). Analogous results to Fig 2 are presented in Fig 3. The F1-score between components detected by OnACID-E and CNMF-E was 0.899. The median correlation between the temporal traces of neurons detected by both implementations was 0.847 ± 0.022.

**Fig 4:**
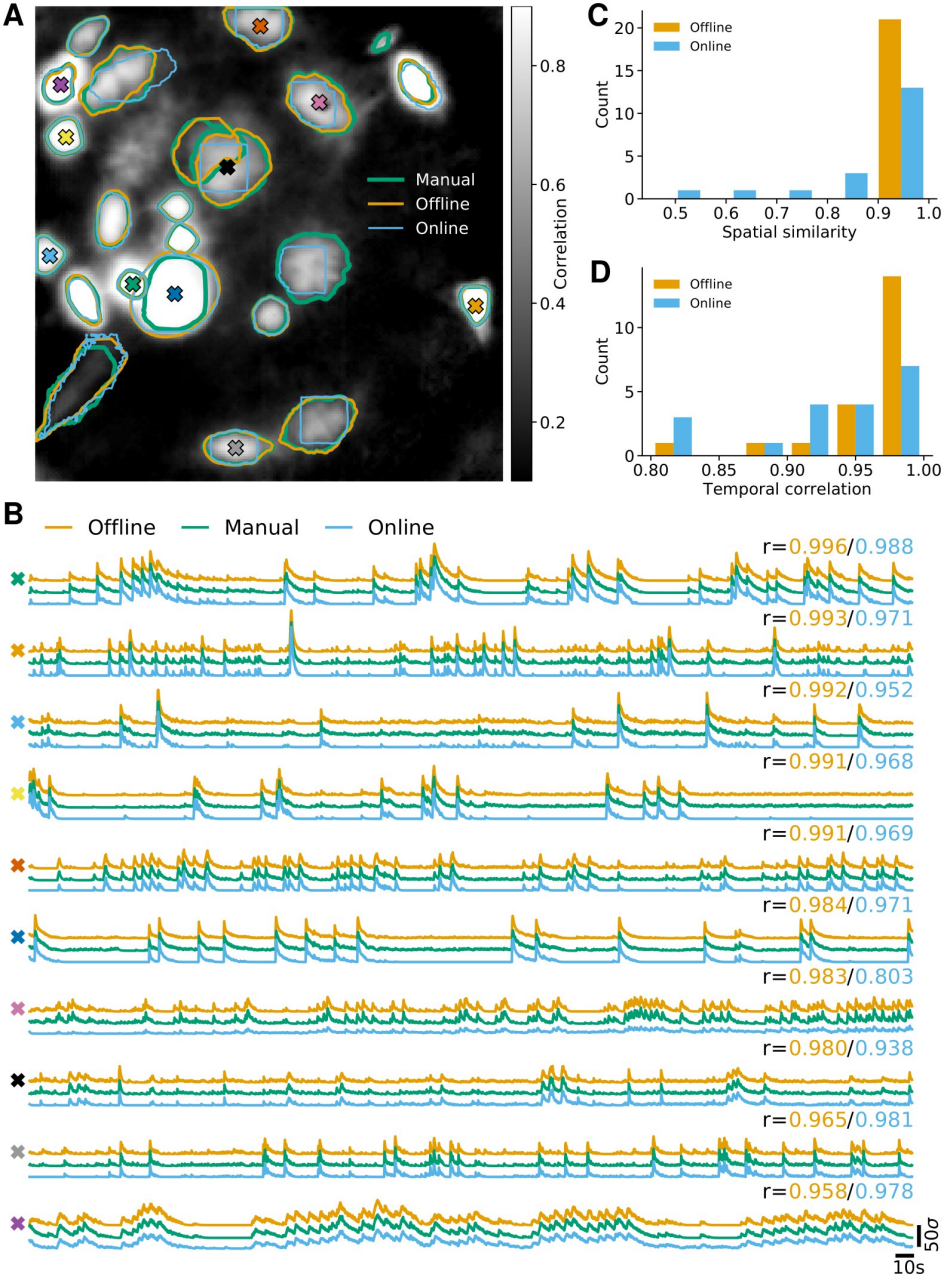
Comparison of OnACID-E with CNMF-E on data from neurons expressing GCaMP6f recorded *in vivo* in mouse ventral hippocampus. **(A)** Contour plots of ground truth neurons (green) as well as all neurons detected by CNMF-E (orange) and OnACID-E (blue), overlaid over the local cross-correlation image of the background-subtracted video data, with background estimated using CNMF-E with manual labeling. Colored symbols match the example traces shown in **(B)**, which illustrate the temporal components of ten example neurons detected by both implementations. The numbers to the upper right of each trace shows the correlation *r* between ‘offline’/ ‘online’ and ‘manual’. **(C)** Histogram of cosine similarities between inferred and true neural shapes. **(D)** Histogram of correlations between inferred and true neural fluorescence traces.

As third analysis we considered data from neurons expressing GCaMP6f recorded *in vivo* in mouse ventral hippocampus, cf. Fig 4. This third dataset consisted of 9000 frames at 15 Hz resolution (for further details refer to [6]). Here the FOV contained few enough neurons to label them manually, although one should be aware that in general human labelling is not perfect with different labelers often differing in their assessment [17]. We detected 23 neurons manually, whereas CNMF-E and OnACID-E detected 21 of these without any additional false positives (Fig 4A), yielding a F1-score of 0.955 when comparing either with the manually labeled components. Testing the quality of the inferred traces is more challenging due to the unavailability of ground truth data. As approximation to ‘ground truth’ we ran CNMF-E initialized with the centers of the manual annotation. We show ten example temporal traces in Fig 4B for CNMF-E, manually seeded CNMF-E and OnACID-E. The cosine similarity $\frac{a^{⊤}a^{*}}{∥a∥∥a^{*}∥}$ between the neural shape obtained with manual initialization a* and inferred neural shape ***a*** is reported in Fig 4C. The correlation between the corresponding temporal traces is shown in Fig 4D. Unsurprisingly, the traces obtained with the offline algorithm are more similar to the ‘ground truth’ traces than the traces obtained with the online algorithm, because the ‘ground truth’ traces were obtained by running the offline CNMF-E algorithm, but with manual instead of automatic initialization. The median correlation between the temporal traces of neurons detected by CNMF-E and ‘ground truth’ was 0.983 ± 0.005, for OnACID-E it was 0.938 ± 0.020.

**Fig 5:**
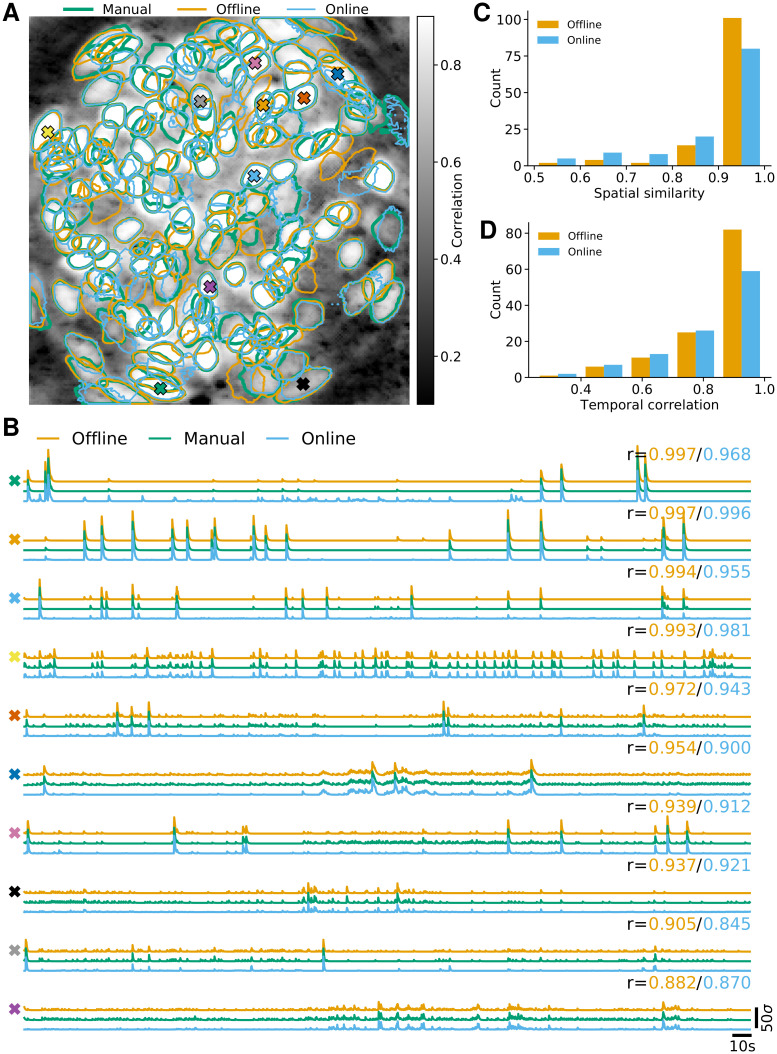
Comparison of OnACID-E with CNMF-E on data from neurons expressing GCaMP6s recorded *in vivo* in mouse bed nucleus of the stria terminalis (BNST). **(A)** Contour plots of ground truth neurons (green) as well as all neurons detected by CNMF-E (orange) and OnACID-E (blue), overlaid over the local cross-correlation image of the background-subtracted video data, with background estimated using CNMF-E with manual labeling. Colored symbols match the example traces shown in **(B)**, which illustrate the temporal components of ten example neurons detected by both implementations. The numbers to the upper right of each trace shows the correlation *r* between ‘offline’/ ‘online’ and ‘manual’. **(C)** Histogram of cosine similarities between inferred and true neural shapes. **(D)** Histogram of correlations between inferred and true neural fluorescence traces.

We repeated the analysis on data from neurons expressing GCaMP6s recorded *in vivo* in mouse bed nucleus of the stria terminalis (BNST). This fourth dataset consisted of 4500 frames at 10 Hz resolution (for further details refer to [6]). We labeled 139 neurons manually, CNMF-E detected 126 of those and 16 additional components, OnACID-E detected 126 of the manually labeled ones and 15 additional components. Analogous results to Fig 4 are presented in Fig 5. The F1-score between components detected by CNMF-E and ‘ground truth’ was 0.897, for OnACID-E it was 0.904. The median correlation between the temporal traces of neurons detected by CNMF-E and ‘ground truth’ was 0.912 ± 0.017, for OnACID-E it was 0.854±0.026. A summary of the characteristics and results for each dataset is given in Table 2.

**Table 2:** Dataset characteristics and performance measures.

Dataset		Striatum	PFC	Hippocampus	BNST
Size (*x* × *y* × *t*)		256 × 256 × 6000	175 × 184 × 9000	200 × 200 × 9000	199 × 203 × 4500
Rate [Hz]		10	15	15	10
F1-Score	CNMF-E	0.891	0.899	0.955	0.897
	OnACID-E			0.955	0.904
Correlation	CNMF-E	0.852 ± 0.008	0.847 ± 0.022	0.983 ± 0.005	0.912 ± 0.017
	OnACID-E			0.938 ± 0.020	0.854 ± 0.026
N	CNMF-E	582	171	21	142
	OnACID-E	554	185	21	141
% of frames processed in real-time	Tracking	100	100	100	100
	OnACID-E	84	94	90	92
	Ring-CNN	100	100	100	100

Individual entries for CNMF-E and OnACID-E denote comparison to ‘ground truth’ components (F1-Score) and traces (correlation) obtained by manual initialization of CNMF-E, shared entries denote direct comparison between CNMF-E and OnACID-E. While all methods process the datasets faster than real time *on average*, only for Tracking and Ring-CNN is the processing speed for *each* frame above the acquisition rate, whereas OnACID-E processes a high percentage of individual frames in real time.

**Fig 6:**
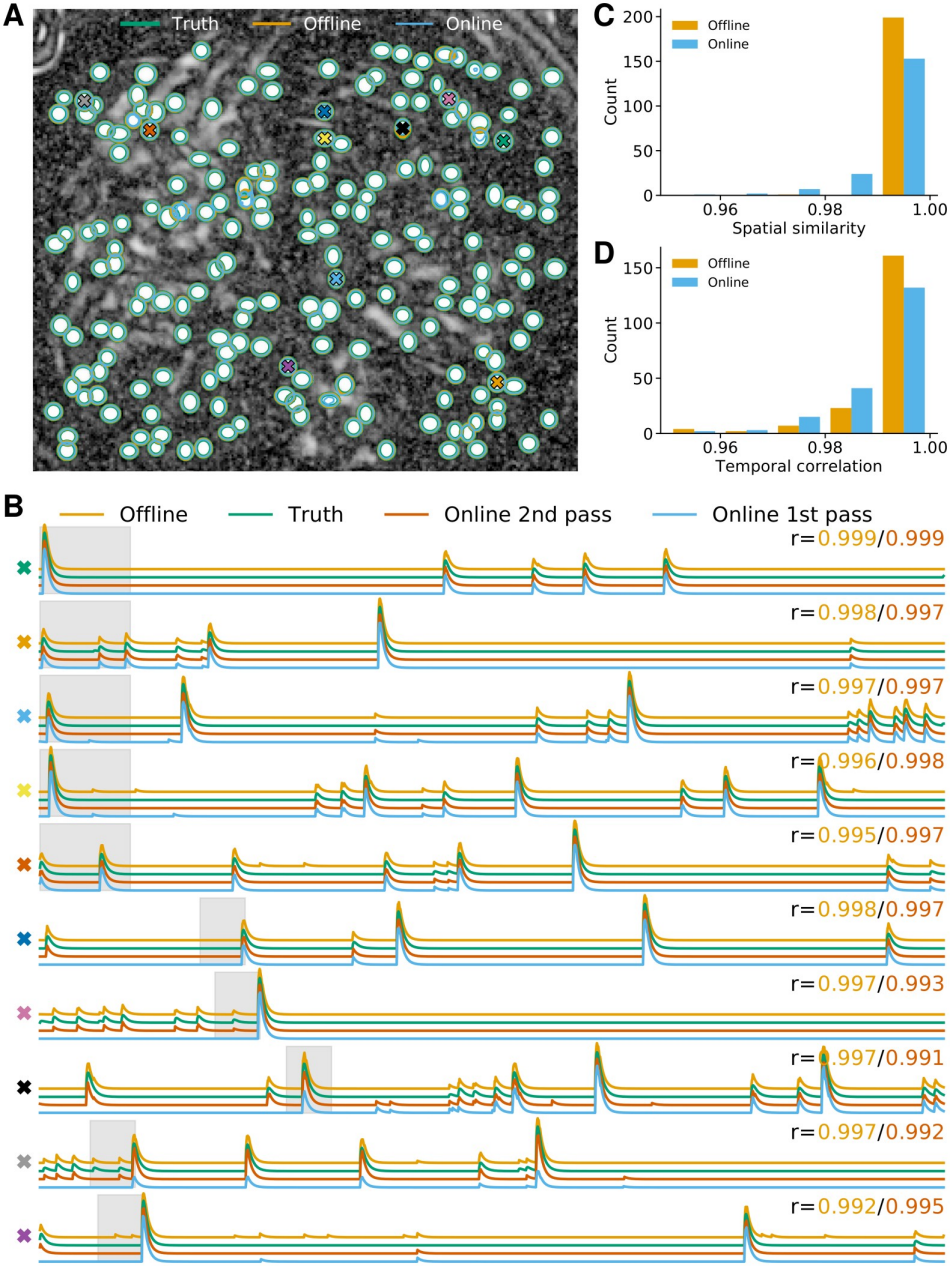
OnACID-E performs similar to CNMF-E in extracting individual neurons’ activity from simulated data. **(A)** Contour plots of ground truth neurons (green) as well as all neurons detected by CNMF-E (orange) and OnACID-E (blue), overlaid over the local cross-correlation image. Colored symbols match the example traces shown in **(B)**, which illustrate the temporal components of ten example neurons detected by both implementations. The first five have been detected in the initialization phase, the last five during online processing. The gray shaded area shows the mini-batch OnACID-E used for the cell’s initialization, thus the area’s right border indicates at what frame the cell was initialized. The numbers to the upper right of each trace shows the correlation *r* between ‘offline’/‘online 2nd pass’ and ground truth. **(C)** Histogram of cosine similarities between inferred and true neural shapes. **(D)** Histogram of correlations between inferred and true neural fluorescence traces.

We also performed the comparison on the simulated data from [6], in order to compare not only the offline and online method with each other but both with underlying ground truth, see Fig 6. Both implementations detect all components (Fig 6A) with a perfect F1-score of 1. We again show ten example temporal traces in Fig 6B. The cosine similarity between true and inferred neural shape is reported in Fig 6C. While CNMF-E tends to capture the neural footprints more accurately, the inferred temporal components (that would be used in the subsequent analysis and are hence more important) are of similar quality, as the correlations with ground truth reveal (Fig 6D). The median correlation between the temporal traces of neurons detected by CNMF-E and ground truth was 0.9961 ± 0.0002, for OnACID-E it was 0.9932 ± 0.0005.

### Computational performance of OnACID-E

We examined the performance of OnACID-E in terms of processing time and memory requirements for the analyzed dorsal striatum dataset (Fig 2) presented above. For the batch as well as the online algorithm we used the Python implementations provided by or added to CaImAn [17], respectively. OnACID-E has very limited memory requirements and can readily be run on a laptop. Thus, unless otherwise mentioned, the analysis was run on a laptop (MacBook Pro 13”, 2017) with Intel Core i7-7567U CPU at 3.5 GHz (2 cores) and 16 GB of RAM running macOS Catalina.

**Fig 7:**
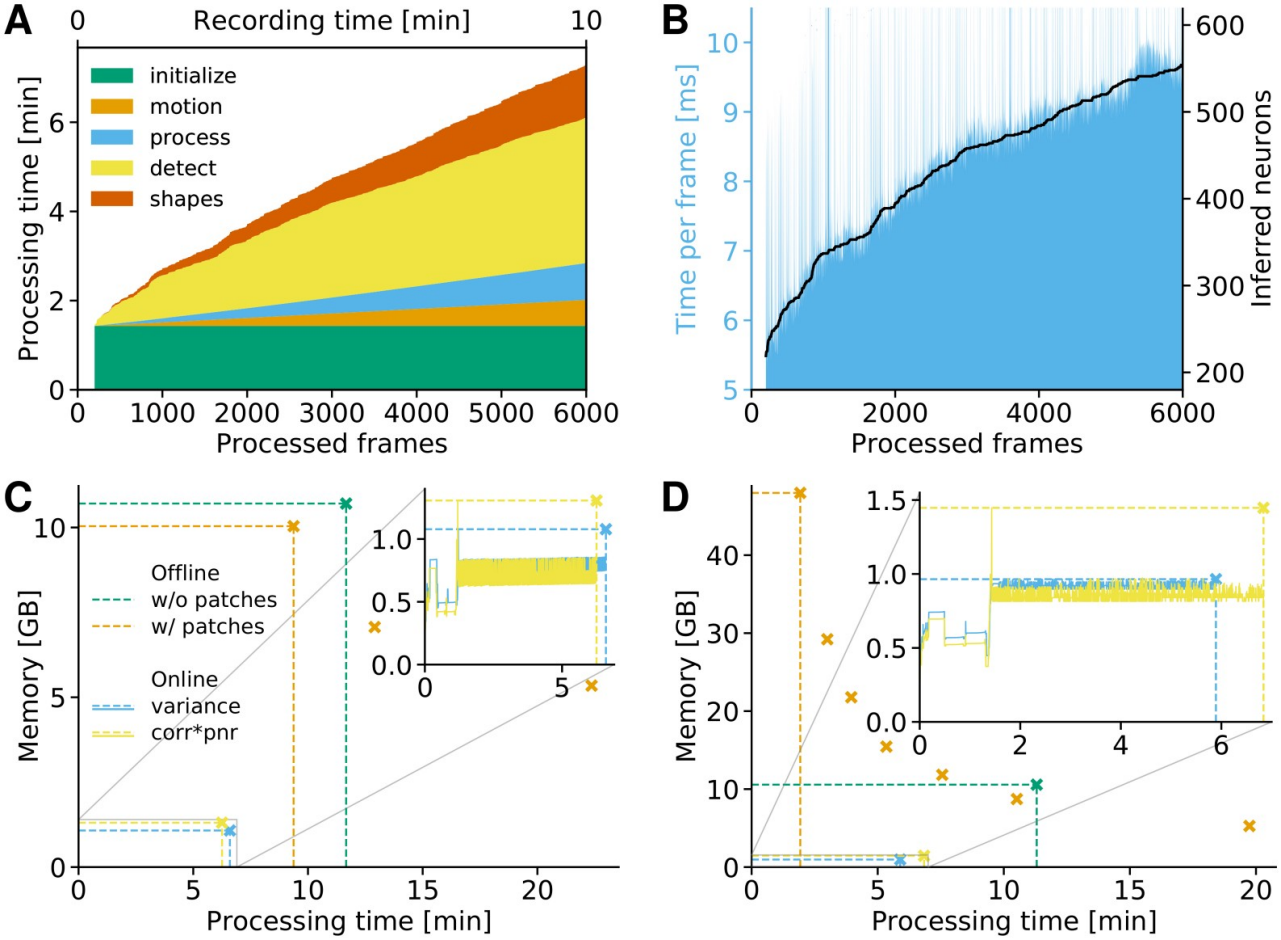
Computing resources of OnACID-E. The same dorsal striatum dataset from Fig 2 consisting of 6000 frames with a 256 × 256 FOV was used. **(A)** Cumulative processing time, separated by time for initialization (occurred only at the beginning), motion correction, tracking existing activity, detecting new neurons, and updating spatial footprints as well as background. **(B)** Cost of tracking neurons’ activity scales linearly with the number of neurons. **(C)** Memory consumption of OnACID-E and CNMF-E. Markers and dashed lines indicate peak memory and overall processing time. Solid lines in the inset depict memory as function of time, and show that the required memory does not increase with the number of recorded frames. Offline processing using CNMF-E was performed with or without patches, online processing using OnACID-E with variance or corr⊙pnr summary image, cf. Methods. The orange markers show peak memory and overall processing time when the number of parallel processes is varied (4, 2 and 1), illustrating the time-memory trade off when processing in patches (more processes can lead to faster processing at the expense of additional memory requirements). **(D)** Analogous results as in (C) when using a single cluster node instead of the laptop, which enabled to process all patches simultaneously. Orange markers show peak memory and overall processing time for 16, 8, 6, 4, 3, 2 and 1 parallel processes.

The processing time of OnACID-E depends primarily on (i) the computational cost of tracking the temporal activity of discovered neurons, (ii) the cost of detecting and incorporating new neurons, and (iii) the cost of periodic updates of spatial footprints and background. Additionally, there is the one-time cost incurred for initialization. Fig 7A and S3(A) Fig show the cost of each of these steps for one epoch of processing. Initialization was performed by running CNMF-E on the first 200 frames, hence the sudden jump at 200 processed frames in Fig 7A. The cost of detecting and incorporating new components remains approximately constant across time and is dependent on the number of candidate components at each time step. In this example three candidate components were used per frame. As noted in [17], a higher number of candidate components can lead to higher recall in shorter datasets at a moderate additional computational cost (see S4 Fig).

The cost of tracking components can be kept low due to simultaneous vectorized updates, and increases only mildly over time as more components are found by the algorithm, cf. Fig 7B and S3(B) Fig. Finally, it is particularly noteworthy that the total processing time was smaller than the duration of the recording. If imaging was performed at a frame rate that is higher by some factor *x*, the cost of tracking would increase by this factor *x*, whereas the periodic updates of spatial footprints and background would still be performed every few seconds (20 s in our case). To keep the time spent on detecting new components invariant one can look for new components every *x*th frame. Thus the total required time is the sum of a high constant cost and the time for tracking that increases linearly with frame rate but is low.

Fig 7C shows the memory usage as function of processing time and compares to CNMF-E with or without splitting the FOV into patches. Sixteen patches of size 96x96 were used and processed in parallel. Due to the limited resources of the laptop (four threads, 16 GB RAM) not all, but merely up to four of the total sixteen patches, could be processed simultaneously in parallel. Fig 7C shows that whereas processing in patches was marginally faster and less memory consuming than processing the entire FOV, both are clearly outperformed with regard to computing time and memory requirements by OnACID-E. It required less memory than the size of the whole data, here 1.5 GB (for single-precision float), and about an order of magnitude less memory than CNMF-E. These results would be even more pronounced for longer datasets lasting not just minutes but hours because the memory consumption remains nearly constant as time progresses and is thus independent of the number of recorded frames.

Fig 7D repeats the analysis of Fig 7C, but using a single node of a linux-based (CentOS) cluster with Intel Xeon Platinum 8168 CPU at 2.7 GHz (24 cores) and 768 GB of RAM. This enabled to process all sixteen patches simultaneously in parallel. Processing can be faster using patches, however, this gain comes at the cost of high memory requirements compared to the raw data size and necessitates a powerful computing environment. These requirement can be mitigated at the expense of longer processing times by processing not all patches in parallel, as the additional orange markers in Fig 7D for 8, 6, 4, 3, 2 and 1 parallel processes show. Online processing on the cluster node took about the same time as on the laptop. OnACID-E strikes the best balance between memory consumption and processing time, making it in particular suitable for processing of long datasets without the need for high performance hardware.

### Performance of the ring CNN approach

For comparison purposes we also tried the online analysis of the same dorsal striatum dataset, using the Ring-CNN background model (Eq 22) with two kernels of width 5 pixels. The model was trained on the first 500 frames (400 frames for training and 100 for validation) using a quantile loss function (Eq 26) with *q* = 0.02, using stochastic gradient descent with the ADAM optimizer. After initialization every frame was passed through the learned model to remove its background and was subsequently processed using the OnACID algorithm [17] with a rank-2 background, cf. Alg 2. During this phase, the background model was kept constant with no additional training, which resulted in faster processing. This was possible because the background model had already converged to a stable value during initialization because of the smaller number of parameters needed to be learned due to the large level of weight sharing. Moreover, the increased width of the filter increased the statistical power of the model making it less sensitive to outliers, and thus aiding faster convergence. Three epochs were used to process the dataset, with the third epoch being used only to track the activity of the existing neurons (and not to detect new components). After the online processing was done, the identified components were merged, and then screened for false positive using the tests employed in the CaImAn package [17].

**Fig 8:**
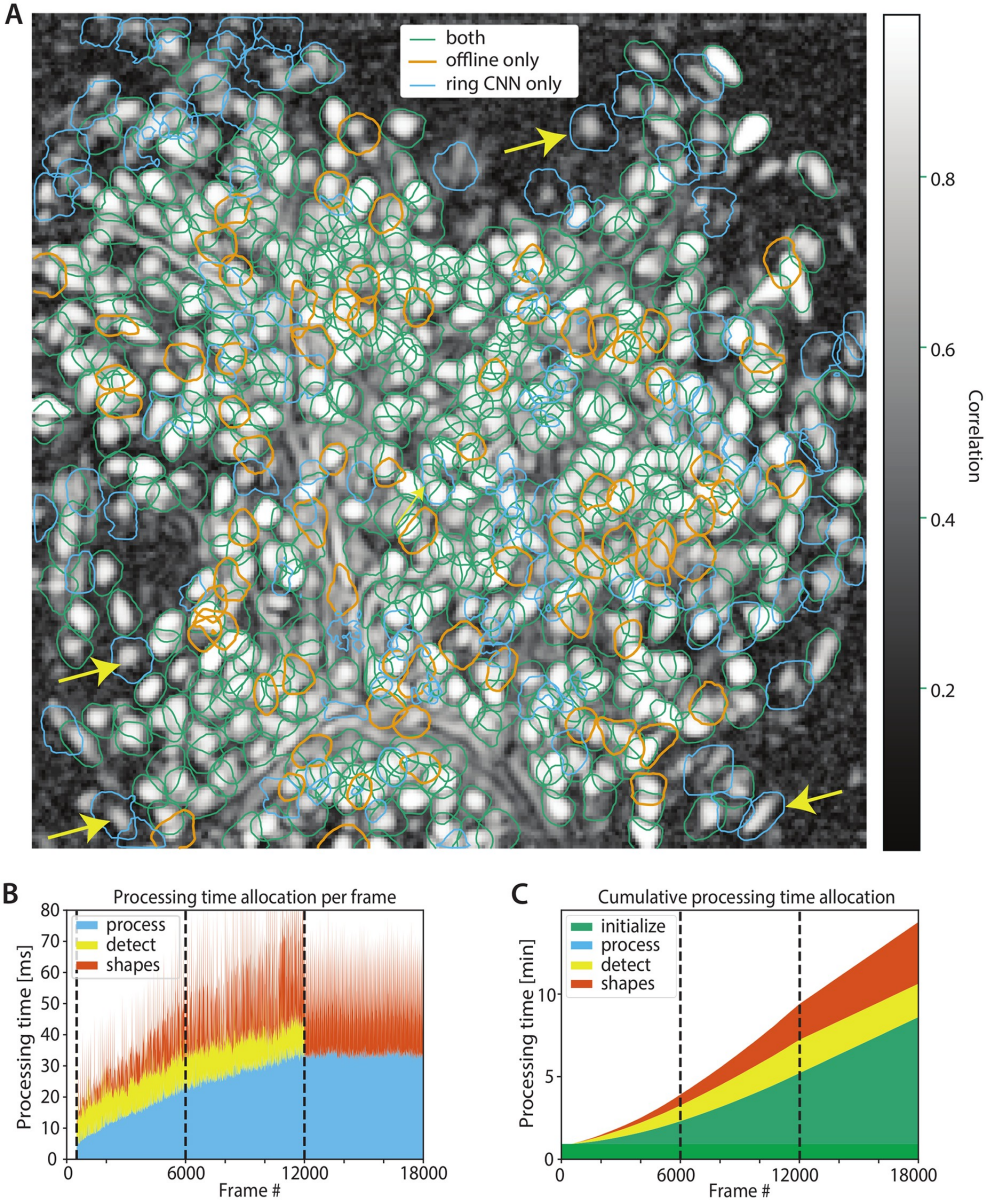
Performance of online approach using a ring CNN background model on the dorsal striatum dataset. **(A)** Contour plots of all neurons detected by the ring CNN approach and CNMF-E using patches overlaid over the local cross-correlation image. The two approaches have a high level of similarity (green contours, F1-score 0.845), with several components identified only by one algorithm (orange contours, CNMF-E only, blue contours, ring CNN only). At least some of the contours identified only by the ring CNN model appear to correspond to actual neurons (yellow arrows). Processing speed per frame **(B)** and cumulatively **(C)** for the ring CNN approach. Dashed lines indicate 1st, 2nd and 3rd epoch. By reducing the background extraction to a simple, GPU-implementable, filtering operation and estimating it only during initialization, the ring CNN approach can achieve high processing speeds for every frame **(B)**, and run a complete epoch on the data faster than OnACID-E **(C)**, cf. Fig 7A. Moreover, it can distribute the computational load evenly amongst all frames making it useful for real time applications.

Lacking a “ground truth” benchmark we compared its performance against the CNMF-E algorithm [6]. The results of the analysis are summarized in Fig 8. The algorithms displayed a high level of agreement (green contours in Fig 8A) with F1-score 0.848 (precision 0.81 and recall 0.888 treating the CNMF-E predictions as “ground truth”). While the agreement between the ring CNN appoach and CNMF-E was lower compared to the agreement between OnACID-E and CNMF-E, this cannot be readily interpreted as underperformance of the ring CNN approach. For example, the ring CNN approach identified several components that have a clear spatial footprint in the correlation image of the spatially filtered data (some examples are highlighted by the yellow arrows).

The computational performance of the ring CNN approach is shown in Fig 8B and 8C. In addition to a computing cluster node, an NVIDIA Tesla V100 SXM2 32GB GPU was deployed to estimate the background model and subsequently apply it. Overall initialization on the 500 frames required around 53s, roughly equally split between estimating and applying the background model, and performing “bare initialization” [17] on the background extracted to find 50 components and initialize the rank-2 background. After that processing was very fast for every frame (Fig 8B) with no computational bottlenecks (as opposed to OnACID-E where updating the background can take significant resources). Overall, the first epoch of processing was completed in 210s (Fig 8C), a factor of 2 improvement over OnACID-E (even without background updating for OnACID-E). A closer comparison between Figs 7A and 8C indicates that the ring CNN approach is faster than OnACID-E for detecting new components, but slower during “tracking”. The reason for that, is that “tracking” in the ring CNN approach includes the background removing step (which requires data transfer to and from the GPU). However, once this step is done, no additional background treatment is required, which speeds up the detection step significantly. More importantly, this allowed a distributed update of shapes amongst all frames (Fig 8B) which kept the processing speed for *each* frame above the acquisition rate of 10Hz, thus achieving real time processing. Since the initialization step can be performed in mini-batches the GPU memory requirements remain limited. After that, online processing is deployed on a frame by frame basis which keep the memory requirements at similar levels compared to OnACID-E (S5 Fig).

**Fig 9:**
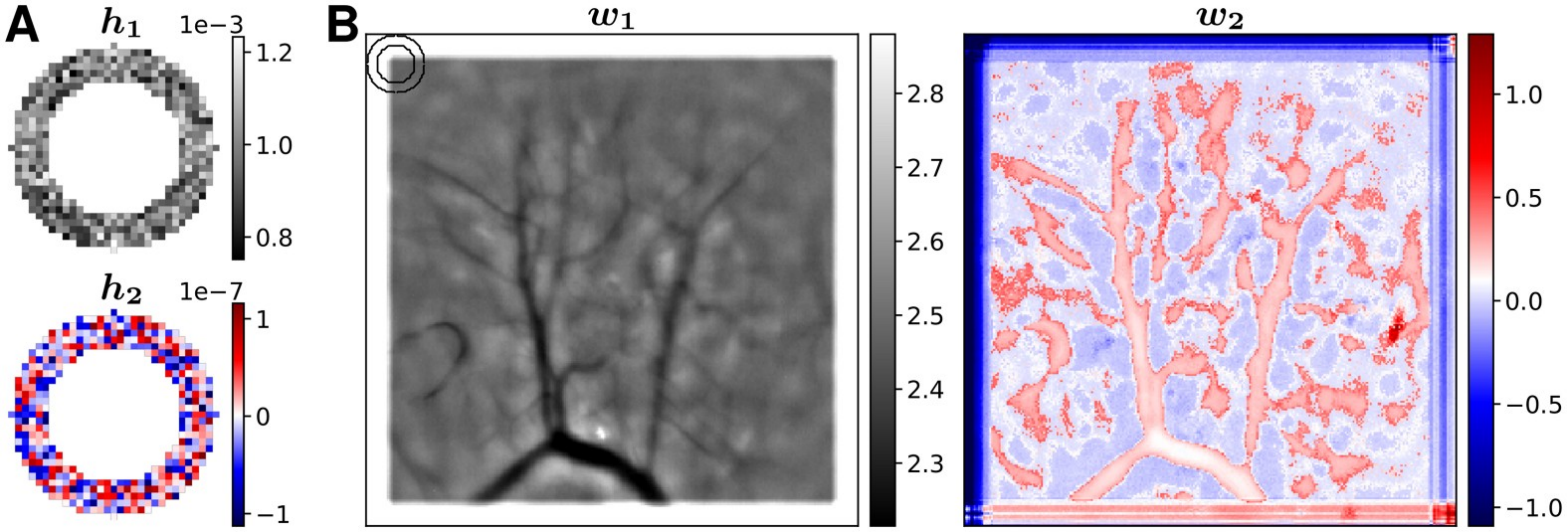
Parameters of the trained ring-CNN for the dorsal striatum dataset. **(A)** Convolution kernels of the first layer. **(B)** Pixel dependent weights of the second layer. The ring in the upper left corner indicates the size of the convolution kernels.

Fig 9 shows the parameters of the trained ring CNN. As described in the Methods, the output of the network is invariant to rotations of the parameters. We used singular value decomposition, such that ***h***_1_, ***w***_1_ correspond to the left and right singular vectors of the larger singular value (14.1), and ***h***_2_, ***w***_2_ to the singular vectors of the smaller singular value (7.46 × 10^−5^). The first convolutional kernel ***h***_1_ shows the typical ring, and the weights ***w***_1_ mostly capture pixel dependent differences in brightness, that are for example due to blood vessels. The second convolutional kernel ***h***_2_ shows deviations from the typical ring, and the weights ***w***_2_ where those deviations are applied. The much lower numerical values of ***h***_2_ and ***w***_2_ compared to *h*_1_ and ***w***_1_ reveal, that here the point spread function (PSF) was largely invariant over the entire FOV. In cases where the PSF varies when compared across the full FOV the number of rings *K* can be adjusted to the diversity of local environments.

**Fig 10:**
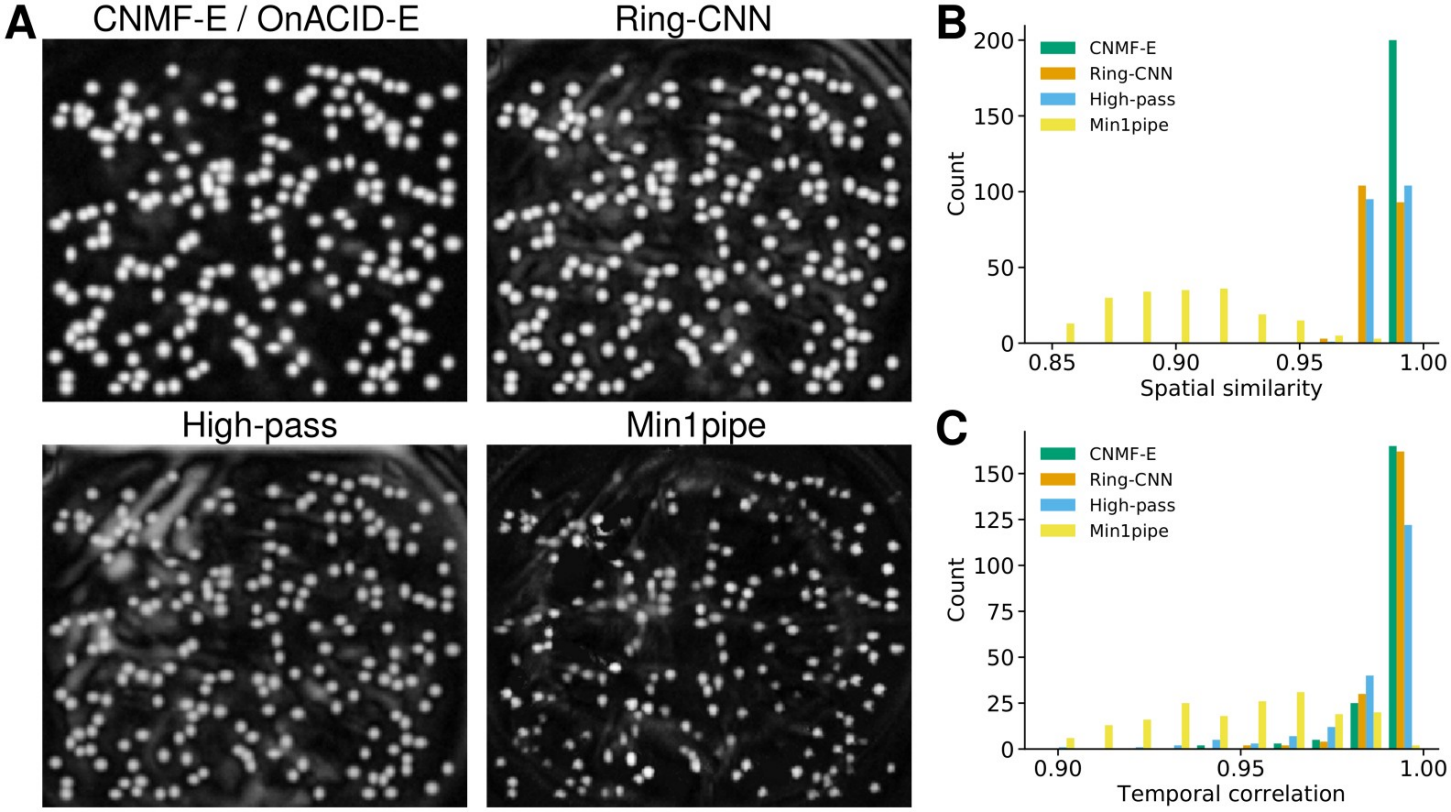
Comparison of background models on simulated data. **(A)** Local cross-correlation images of the background-subtracted video data. **(B)** Histogram of cosine similarities between inferred and true neural shapes. **(C)** Histogram of correlations between inferred and true neural fluorescence traces.

The ring CNN approach introduced a new background model that was inspired by the ring model of CNMF-E/OnACID-E. It relies on removing the cumbersome background first, followed by an algorithm that has already been established for 2-photon data such as CNMF or OnACID. Similarly, Min1pipe [7] also relies on turning the imaging data into a stack of background-free frames as the first step, but uses morphological opening to estimate the background. In order to compare these methods, that use different background models, to underlying ground truth we considered again the simulated data from [6]. As a simple baseline we also included spatial high-pass filtering using a second order Butterworth filter with cut-off frequency of 6 inverse pixels. Fig 10A shows the local cross-correlation image of the data after removing the background for the four background models. While each method bring out all neurons that are not, or barely, visible in the raw video data (Fig 3 in [6]), the background model of CNMF-E visually captures the true background best. However, a good background estimate is just a means to enable extraction of accurate temporal traces that would be used in the subsequent analysis and are hence most important. To investigate how the background model influences the latter, we ran CNMF, initialized with the true neural centers, on these back-ground subtracted data, to obtain the temporal traces and updated spatial footprints. The cosine similarities between true and inferred neural shapes are reported in Fig 10B, with high values for the ring model of CNMF-E/OnACID-E, similar ones for Ring-CNN and high-pass filtering, and lower ones for morphological opening (Min1pipe). The inferred temporal components are of similar quality for CNMF-E and Ring-CNN, as the correlations with ground truth reveal (Fig 10C). The traces obtained with spatial high-pass filtering and morphological opening rank a close third and distant fourth respectively. The median correlation between ground truth and the temporal traces of neurons detected was 0.9970 ± 0.0003 for CNMF-E, 0.9942 ± 0.0004 for Ring-CNN, 0.9914 ± 0.0005 for high-pass filtering, and 0.9460 ± 0.0036 for morphological opening. We repeated the analysis on real data using the dorsal striatum data, cf. S6 Fig. Due to the lack of ground truth we compared to the results obtained with CNMF-E. Again, the Ring-CNN outperforms high-pass filtering.

### Real time processing

**Fig 11:**
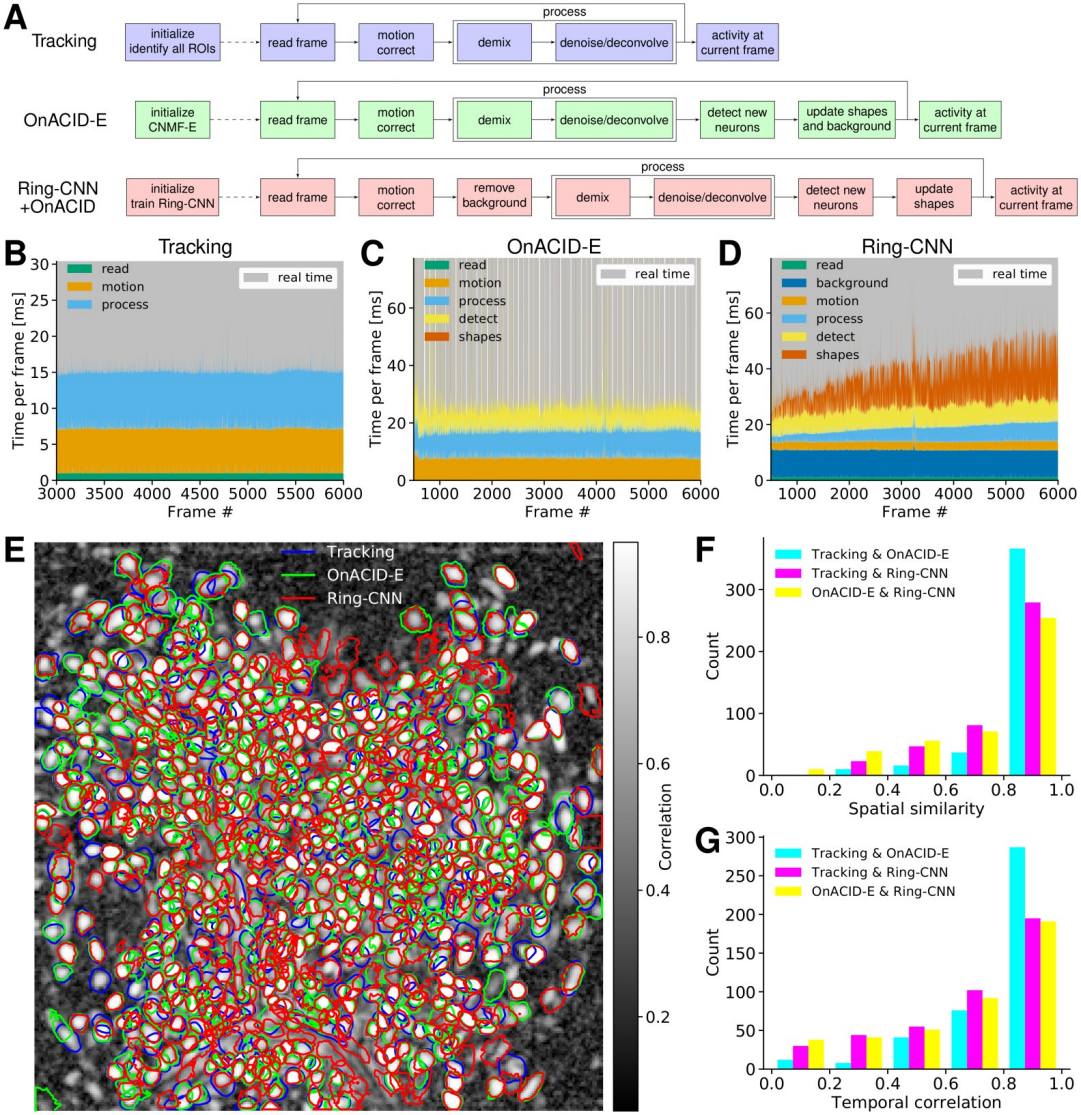
Online processing in real time. **(A)** Flow-charts for three online processing pipelines. **(B-D)** Time per frame for (**B**) tracking pre-identified ROIs, (**C**) OnACID-E, and (**D**) Ring-CNN, separated by time to read the frame, motion correct it, process it (i.e. demix overlapping components and deconvolve temporal trace), and, where applicable (OnACID-E and Ring-CNN), detect new neurons, update spatial footprints, and remove background. The upper limit of the vertical axis was set to twice the average total time per frame. The gray shading indicates the frames that are processed in real time. **(E)** Contour plots of all neurons detected by CNMF-E on a sufficiently long initial batch (Tracking), OnACID-E, and the Ring-CNN approach overlaid over the local cross-correlation image. **(F)** Histogram of pairwise cosine similarities of neural shapes for neurons detected using tracking, OnACID-E, and Ring-CNN. **(G)** Histogram of pairwise correlations of neural fluorescence traces.

One compelling reason to use online processing, not just for data streams but also for already recorded data, is that it circumvents the computational demands of offline processing. Even more impactful is its application to real time processing in closed loop experiments [4], for example combining imaging with optogenetic manipulation [30], which recently became technically possible also for 1-photon microendoscopes [31, 32]. Here we describe three approaches, cf. Fig 11A, for processing microendoscopic data in real time. We again considered the dorsal striatum data (Fig 2) and processed it using the MacBook Pro laptop.

The first, and arguably simplest, approach, denoted as “Tracking”, is to have a sufficiently long initialization phase to identify all ROIs. We processed an initial batch of 3000 frames (5 min) using CNMF-E, though other offline pipelines could likewise be used, to initialize the online method OnACID-E. Once the initial batch has been processed, the real time experiment begins, during which each frame has to be read from the camera, corrected for motion artifacts, and processed to track each neuron’s activity. The latter entails demixing the fluorescence contributions of (potentially overlapping) ROIs and background, as well as denoising/deconvolving each neuron’s temporal trace. Fig 11B breaks down these times for each frame. In an actual real time experiment one would read directly from camera, here we report the time to read the frames from the laptop’s solid-state drive. For further speed ups one could spatially decimate the identified ROIs and the raw data frames, and still accurately recover denoised fluorescence traces and deconvolved neural activity [26].

The second approach, denoted as “OnACID-E”, is to have merely a short initialization phase (we used 500 frames) followed by online processing using OnACID-E that not only tracks the activity of already detected ROIs as the first approach does, but includes automatic detection of new components and updates neural shapes as well as background. The latter updates are performed every few seconds (20 s in our case). They are however so computationally costly that real time performance is lost for this and some subsequent frames, as visible in the periodic pattern of the gray shading in Fig 11C. Thus, instead of the usual waiting for the camera to provide the next image, there are few frames for which the images are acquired faster than processed. Therefore we use a separate computing process to acquire and add the images to a FIFO (First-In-First-Out) queue at regular time intervals. The main process reads the next image from this queue, and waits for it if the queue is empty. Although this approach is not fully real time, 84% of the 5500 frames that have been processed online have actually been processed in real time.

The third approach, denoted as “Ring-CNN”, is similar to the second in using merely a short initialization phase (we used 500 frames), but instead of OnACID-E it uses Ring-CNN and OnACID for online processing. The background removing step involves evaluation of the CNN. This can be expressed as a sparse matrix multiplication, see Eq (23), which is quickly evaluated even on a laptop, cf. Fig 11D. Because the real time experiment starts only after the initial batch has been processed, the CNN can in principle be trained on a laptop as well. However, usage of a GPU is still advisable for this step in order to reduce the time where the camera is idle, i.e. the time between recording the last frame of the initial batch and starting the real-time experiment, which can take about an hour on a laptop, few minutes on a GPU, and even less than a minute on the high-performance GPU used in the previous subsection. Here we reused the previously trained CNN and converted it into a sparse matrix. The CNN possibly generalizes across imaging sessions, but we did not have access to the imaging data of multiple sessions of the same animal to test this. If retraining is necessary, a GPU is needed in praxis, but it does not need to be high end. This approach avoids OnACID-E’s complicated interaction between background and neural shapes, which allows a distributed update of the latter amongst all frames, thus achieving real time performance for each frame.

While all three approaches yield similar results, the first two model the background using the same ring model of CNMF-E, which as expected yields more similar results for them, compared to the third that employs the Ring-CNN. Fig 11E shows that all three approaches detect similar components. When comparing to Tracking, the F1-score was 0.892 for OnACID-E and 0.805 for Ring-CNN. This also holds for the spatial (Fig 11F) and temporal (Fig 11G) similarity measures when performing pairwise comparisons of the three approaches.

## Discussion

We presented an online method to process 1-photon microendoscopic video data. Our modeling assumptions are the same as in the popular offline method CNMF-E; however, our online formulation yields a more efficient yet similarly accurate method for the extraction of in vivo calcium signals. A major bottleneck for processing microendoscopic data has been the amount of memory required by CNMF-E. Our online approach solves this issue since it reduces the memory footprint from scaling linearly with the duration of the recording to being constant. We also provided an additional variant that uses a convolutional based background model that aims to exploit the location invariant properties of the point spread function. This approach enables the estimation of a stable background model by using just an initial portion of the data. As a result, it can lead to faster processing and also be coupled to 2-photon processing algorithms by using this model to remove the background from each frame as a preprocessing step. While the background model is invariant to brightness changes, if the background structure was to change in a more intricate way owing to drug delivery, optogenetic stimulation, or other experimental manipulations, the employed 2-photon processing algorithm, e.g. OnACID, can itself include background components that are capable of capturing fluctuations, or training the CNN could continue based on the incoming data stream.

For detecting centroids of new sources OnACID-E examines a static image. Following [21], such an image can be obtained by computing the variance across time of the spatially smoothed residual buffer. As an additional option to obtain a static summary image we added the computation of peak-to-noise ratio and local cross correlation across time of the residual buffer, following the proposal of [6]. For efficiency, this computation is performed online using incremental updates. While both options work very well in practice, different approaches for detecting neurons in static images or in a short residual buffer could potentially be employed here, e.g. dictionary learning [33], combinatorial clustering [34] or deep neural networks [35, 36]. However, these approaches likely come with higher computational cost, and—having been developed for offline processing—would probably need to be modified for data streams, and in the case of neural networks be retrained.

Similarly to [21], our current implementation screens the candidate components for quality using some quantitative measures and thresholds. For 2-photon data [17] suggested to use a neural net classifier instead for better accuracy. Training a neural network requires labelled data, which is currently not publicly available for 1-photon microendoscopic video data. Once labelled ground truth data is available, a neural network could be trained on it and OnACID-E be readily augmented to use this classifier. Such ground truth data would also enable to thoroughly benchmark different source extraction algorithms and their implementations.

Apart from enabling rapid and memory efficient analysis of microendoscopic 1-photon data, our online pipeline also facilitates closed-loop behavioral experiments that analyze data on-the-fly to guide the next experimental steps or to control feedback. After recording a short initial batch of data for about one minute and processing it to initialize the online method, one can start the closed loop real time processing experiment. Although we did not have access to perform real time analysis hooked to an actual experiment, we emulated the environment to the best of our abilities. The current implementation of OnACID-E is already faster than real time on average. On a per-frame basis the processing speed exceeds the data rate for the majority of frames, and only when the periodic updates of sufficient statistics, shapes, and background are performed can the speed drop below the data rate. In principle, speed gains could be obtained by performing these periodic updates, and computations that occur only sporadically (incorporating a new neuron), in a parallel thread with shared memory. We defer that to future work. This speed drop below the data rate can be ameliorated by using a larger initialization batch for OnACID-E. Once enough initial data has been seen and processed, the computationally expensive search for components as well as the spatial footprint and background updates can be turned off, because all regions of interest have been detected and their shapes as well as the background converged to stable values. Further, as presented, this compromise can be avoided altogether by endowing the background with a convolutional structure that enables faster convergence in the background estimation. This subsequently enables updating of spatial footprints in a distributed sense, while maintaining faster than real time processing rates at *every* frame by keeping the ability to detect and incorporate new components. In summary, the Ring-CNN model lends itself better to actual real-time processing, whereas OnACID-E closely resembles the popular CNMF-E algorithm and lends itself to situations where the computing resources are not sufficient to run CNMF-E, or for real-time processing with sufficiently long initialization phase.

We provide a Python implementation of our algorithm online within CaImAn, an open-source library for calcium imaging data analysis (https://github.com/flatironinstitute/CaImAn) [17]. In order to facilitate the use of the presented algorithms in real time experimental scenarios, we have provided a set of example files and a flexible multi-threaded computational infrastructure which can be adapted to a variety of experimental settings. We have embedded online computations in a thread that is executed in parallel, while data can be incrementally added to a First-in-First-Out thread-safe queue. This software engineering design enables the use of OnACID-E with any type of acquisition systems, ranging from USB camera acquisition to dedicated high speed cameras with optimized hardware interfaces. The final user or camera company only needs to implement a small thread which pipes the frames into the queue.

## Supporting information

S1 Appendix Algorithmic description. Pseudocode for the various steps of the online processing pipeline.
(PDF)

S1 Fig Additional components detected by CNMF-E in the dorsal striatum data from Fig 2. (TIF)

S2 Fig Additional components detected by OnACID-E in the dorsal striatum data from Fig 2. (TIF)

S3 Fig Processing time of OnACID-E using the variance summary image. Analogous plots to Fig 7A and 7B, but using the energy for each pixel of the residual buffer to create the summary image instead of the Corr*PNR summary image (see Methods).
(TIF)

S4 Fig Effect of initial batch size and number of considered candidate components. **(A)** Detected components for different sizes of the initial batch without adjusting other parameters. Components detected in the initial batch are shown as solid contours, those detected during online processing as dashed contours. **(B)** Number of detected components for the initial batch sizes considered in (A). **(C)** Number of detected components for a varying number of candidate components.
(TIF)

S5 Fig Memory usage during real-time processing. Memory and number of neurons as function of processed frames for **(A)** Tracking, **(B)** OnACID-E, and **(C)** Ring-CNN + OnACID.
(TIF)

S6 Fig Comparison of background models on the dorsal striatum data from Fig 2. **(A)** Local cross-correlation images of the background-subtracted video data. **(B)** Histogram of cosine similarities between inferred neural shapes and the ones obtained with CNMF-E. **(C)** Histogram of correlations between inferred neural fluorescence traces and the ones obtained with CNMF-E.
(TIF)

S1 Video Depiction of OnACID-E. Top left: Raw data and cell contours of all until then identified components. Top right: Inferred activity (without background). Bottom left: Corr*PNR summary image (see Methods) and accepted regions for new components (magenta squares). Bottom right: Reconstructed activity.
(MP4)
