# Supplementary material for: Online analysis of microendoscopic 1-photon calcium imaging data streams
Source: PLoS Comput Biol. 2021 Jan 28;17(1):e1008565. doi: 10.1371/journal.pcbi.1008565 (PMC7842953; doi:10.1371/journal.pcbi.1008565)
Supplement: S1 Appendix — Pseudocode for the various steps of the online processing pipeline. (PDF) [file pcbi.1008565.s001.pdf]

Online analysis of microendoscopic 1-photon calcium imaging data streams

Johannes Friedrich[1*], Andrea Giovannucci[2], Eftychios A. Pnevmatikakis[1]

1 Flatiron Institute, Simons Foundation, New York, New York, United States of America
2 Joint Department of Biomedical Engineering, University of North Carolina at Chapel Hill and North Carolina State University; and UNC Neuroscience Center, University of North Carolina at Chapel Hill, Chapel Hill, North Carolina, United States of America

* jfriedrich@flatironinstitute.org

S1 Appendix. Algorithmic description

Here we present in pseudocode the various steps of the online processing pipeline. For ease of exposition, some details and speedup tricks used in the actual implementation have been omitted, such as the online update of the summary image used for neuron detection or the spatial decimation of the background.

Algorithm S1 UPDATETRACES

Require: Spatial footprints matrix $\boldsymbol{A}$, current value of temporal traces $\boldsymbol{c}$, current data frame $\boldsymbol{y}$, groups $\mathcal{G}$, tolerance level ε, precomputed $\boldsymbol{V} = \boldsymbol{A}^\top \boldsymbol{A}$, $\boldsymbol{b}_1 = \boldsymbol{A}^\top(\boldsymbol{W}\bar{\boldsymbol{b}} - \bar{\boldsymbol{b}})$, $\boldsymbol{B}_2 = \boldsymbol{A}^\top \boldsymbol{W}$, $\boldsymbol{B}_3 = \boldsymbol{A}^\top \boldsymbol{W}\boldsymbol{A}$.

$\boldsymbol{u} = \boldsymbol{A}^\top \boldsymbol{y}$
$\boldsymbol{v} = \text{diag}\{\boldsymbol{V}\}$
$\boldsymbol{b}_4 = \boldsymbol{B}_2\boldsymbol{y} - \boldsymbol{b}_1$
$\boldsymbol{c}_{\text{old}} \leftarrow 0$
while $\|\boldsymbol{c} - \boldsymbol{c}_{\text{old}}\| \geq \varepsilon \|\boldsymbol{c}_{\text{old}}\|$ **do**
    $\boldsymbol{c}_{\text{old}} \leftarrow \boldsymbol{c}$
    $\boldsymbol{b}_5 = \boldsymbol{b}_4 - \boldsymbol{B}_3\boldsymbol{c}$ ▷ $\boldsymbol{A}^\top \boldsymbol{B}_{:,t}$
    for $i = 1 \to |\mathcal{G}|$ **do**
        $\boldsymbol{c}[G_i] \leftarrow \left\lfloor \boldsymbol{c}[G_i] + \frac{\boldsymbol{u}[G_i] - \boldsymbol{V}[G_i,:]\boldsymbol{c} - \boldsymbol{b}_5[G_i]}{\boldsymbol{v}[G_i]} \right\rfloor_+$ ▷ (Division is pointwise)
return $\boldsymbol{c}$

References

1. Pnevmatikakis EA, Giovannucci A. NoRMCorre: An online algorithm for piecewise rigid motion correction of calcium imaging data. Journal of Neuroscience Methods. 2017;291:83–94.

Algorithm S2 UPDATESUFFSTATISTICS

Require: buffer of data $\tilde{\boldsymbol{Y}}$, buffer of denoised traces $\tilde{\boldsymbol{C}}$, background weights $\boldsymbol{W}$, constant background $\bar{\boldsymbol{b}}$, spatial footprints $\boldsymbol{A}$, sufficient statistics $\boldsymbol{\chi}, \boldsymbol{L}, \boldsymbol{M}$, buffer length T_p, time step t

$\tilde{\boldsymbol{X}} = \tilde{\boldsymbol{Y}} - \boldsymbol{A}\tilde{\boldsymbol{C}} - \bar{\boldsymbol{b}}\boldsymbol{1}_{T_p}^{\top}$
$\boldsymbol{\chi} \leftarrow \boldsymbol{\chi} + \tilde{\boldsymbol{X}}\tilde{\boldsymbol{X}}^{\top}$ ▷ Update only $\boldsymbol{\chi}[i, \boldsymbol{r}_l(i)]$ and $\boldsymbol{\chi}[\boldsymbol{r}_l(i), \boldsymbol{r}_l(i)]$ $\forall i$ where $\boldsymbol{r}_l(i)$ are pixels on ring around i with radius l
$\hat{\boldsymbol{Y}} = \tilde{\boldsymbol{Y}} - \boldsymbol{W}\tilde{\boldsymbol{X}} - \bar{\boldsymbol{b}}\boldsymbol{1}_{T_p}^{\top}$
$\boldsymbol{L} \leftarrow \frac{t-T_p}{t}\boldsymbol{L} + \frac{1}{t}\hat{\boldsymbol{Y}}\tilde{\boldsymbol{C}}^{\top}$ ▷ Update only $\boldsymbol{L}[\boldsymbol{p}(n), n]$ $\forall n$ where $\boldsymbol{p}(n)$ is the 'support' of $\boldsymbol{A}[:, n]$
$\boldsymbol{M} \leftarrow \frac{t-T_p}{t}\boldsymbol{M} + \frac{1}{t}\tilde{\boldsymbol{C}}\tilde{\boldsymbol{C}}^{\top}$
return $\boldsymbol{\chi}$, $\boldsymbol{L}$, $\boldsymbol{M}$

Algorithm S3 UPDATESHAPES

Require: Sufficient statistics $\boldsymbol{L} = (\boldsymbol{Y} - \boldsymbol{B})\boldsymbol{C}^{\top}, \boldsymbol{M} = \boldsymbol{C}\boldsymbol{C}^{\top}$, current value of spatial footprints $\boldsymbol{A}$, maximum number of iterations m_{iter}, number of components N

for iter $= 1 \rightarrow m_{\text{iter}}$ **do**
  for $n = 1 \rightarrow N$ **do**
    $\boldsymbol{p} = \text{find}(\boldsymbol{A}[:, n] > 0)$ ▷ Find the pixels where component n can be non-zero
    $\boldsymbol{A}[\boldsymbol{p}, n] \leftarrow \left\lfloor \boldsymbol{A}[\boldsymbol{p}, n] + \frac{\boldsymbol{L}[\boldsymbol{p}, n] - \boldsymbol{A}[\boldsymbol{p}, :]\boldsymbol{M}[:, n]}{\boldsymbol{M}[n, n]} \right\rfloor_{+}$
return $\boldsymbol{A}$

Algorithm S4 UPDATEBACKGROUND

Require: Sufficient statistics $\boldsymbol{\chi} = \boldsymbol{X}\boldsymbol{X}^{\top}$, ring radius l, number of pixels d

for $i = 1 \rightarrow d$ **do**
  $\boldsymbol{r}_l(i) = \text{ring}(i, l)$ ▷ Get pixels on ring around i with radius l
  $\boldsymbol{W}[i, \boldsymbol{r}_l(i)] = \boldsymbol{\chi}[i, \boldsymbol{r}_l(i)]\boldsymbol{\chi}[\boldsymbol{r}_l(i), \boldsymbol{r}_l(i)]^{-1}$
return $\boldsymbol{W}$

Algorithm S5 DetectNewComponents

Require: Spatial footprints matrix $\boldsymbol{A}$, temporal traces matrix $\boldsymbol{C}$, background weights $\boldsymbol{W}$, constant background $\bar{\boldsymbol{b}}$, current number of components N, current state of groups $\mathcal{G}$, current residual buffer $\boldsymbol{R}_{\text{buf}}$, current data frame $\boldsymbol{y}$. Parameters: neuron size τ, threshold for correlation in space r_s, threshold for correlation in time r_t.

```
repeat = True
x = y − AC[:, end] − b̄
R_buf ← [R_buf[:, 2 : l_b], x − Wx]                      ▷ Update residual buffer
while repeat do
    e ← ComputeSummaryImage(R_buf)
    (i_x, i_y) = arg max e                                 ▷ Find the point of maximal value
    N_(i_x,i_y) = {(x, y) : |x − i_x| ≤ τ, |y − i_y| ≤ τ}   ▷ Define a neighborhood around (i_x, i_y)
    [a_new, c_new] = NMF(R_buf[N_(i_x,i_y), :], 1)          ▷ Perform a local rank-1 NMF
    r = Corr(a_new, Mean(R_buf))                           ▷ Compute correlation coefficient in space
    o = Find(a_new^⊤ A[N_(i_x,i_y), :] > 0)                 ▷ Find components that overlap
    if ∃n ∈ o : Corr(c_new, C[n, t − l_b + 1 : t]) > r_t then
        r ← 0                                              ▷ Detect possible duplicates and stop procedure
    if r > r_s then                                        ▷ New component is accepted
        Zero-pad a_new and c_new to match dimensionality
        N ← N + 1
        G ← JoinGroups(A, G, a_new)
        A ← [A, a_new]
        C ← [C; c_new]
        R_buf ← R_buf − a_new c_new
    else
        repeat = False
return A, C, N, G, R_buf
```

Algorithm S6 AlignFrame

Require: Current data frame $\boldsymbol{y}_t$, rolling buffer of background and trace values $\boldsymbol{b}_s, \boldsymbol{c}_s, s = t-M, \dots, t-1$ spatial footprints $\boldsymbol{A}$, high pass spatial filter H, rest of parameters.

```
y_t^f = h ∗ y_t                                             ▷ High pass filtering of data
m = h ∗ ( (1/M) Σ_{s=t−M}^{t−1} (b_s + A c_s) )             ▷ Template construction
T = NoRMCorre(y_t^f, m)                                     ▷ Find alignment [1]
y_t ← T(y_t)                                                ▷ Tranformation application
return y_t
```
