# Supplementary figures and images for: Online analysis of microendoscopic 1-photon calcium imaging data streams
Source: PLoS Comput Biol. 2021 Jan 28;17(1):e1008565. doi: 10.1371/journal.pcbi.1008565 (PMC7842953; doi:10.1371/journal.pcbi.1008565)

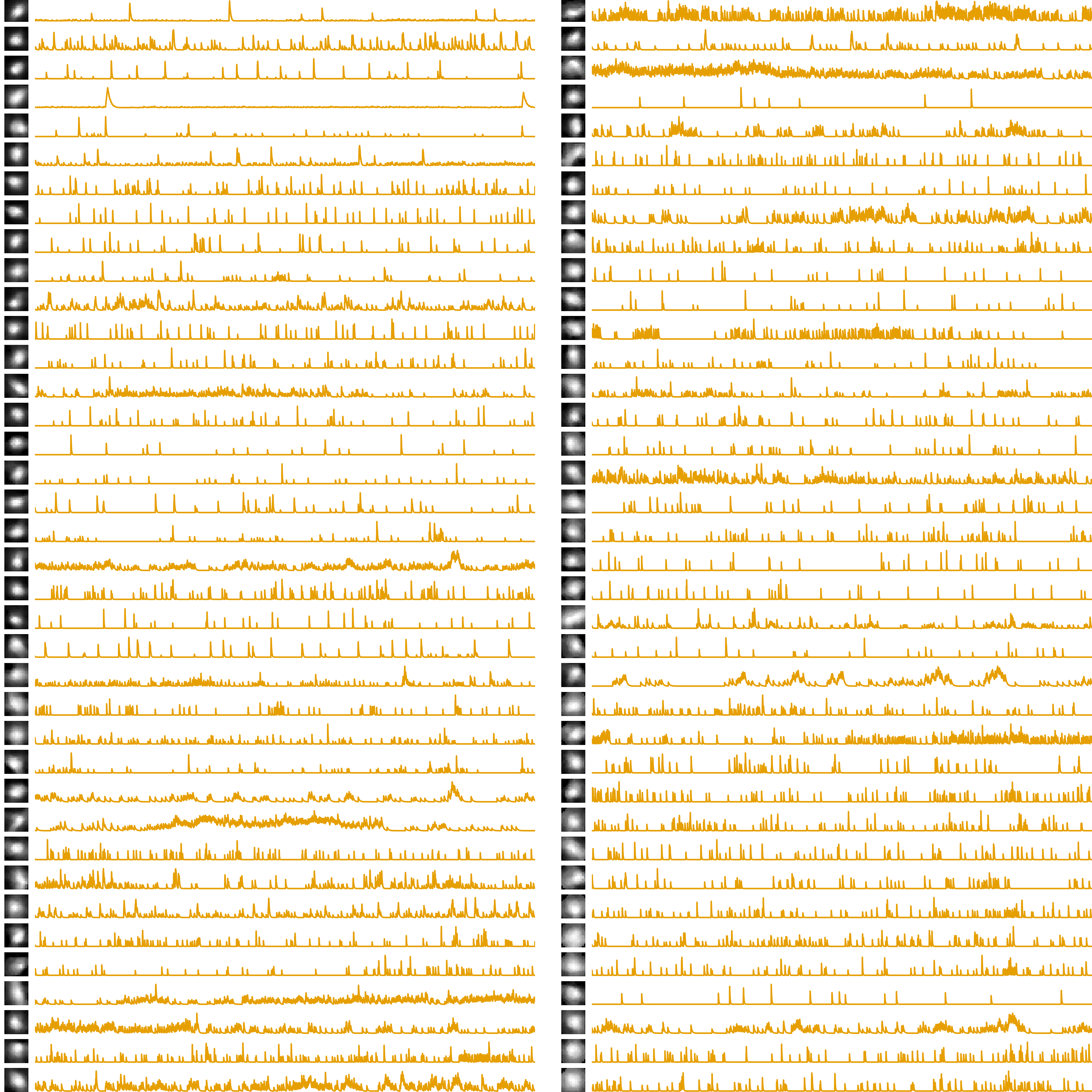

Supplement: S1 Fig — (TIF) [file pcbi.1008565.s002.tif]

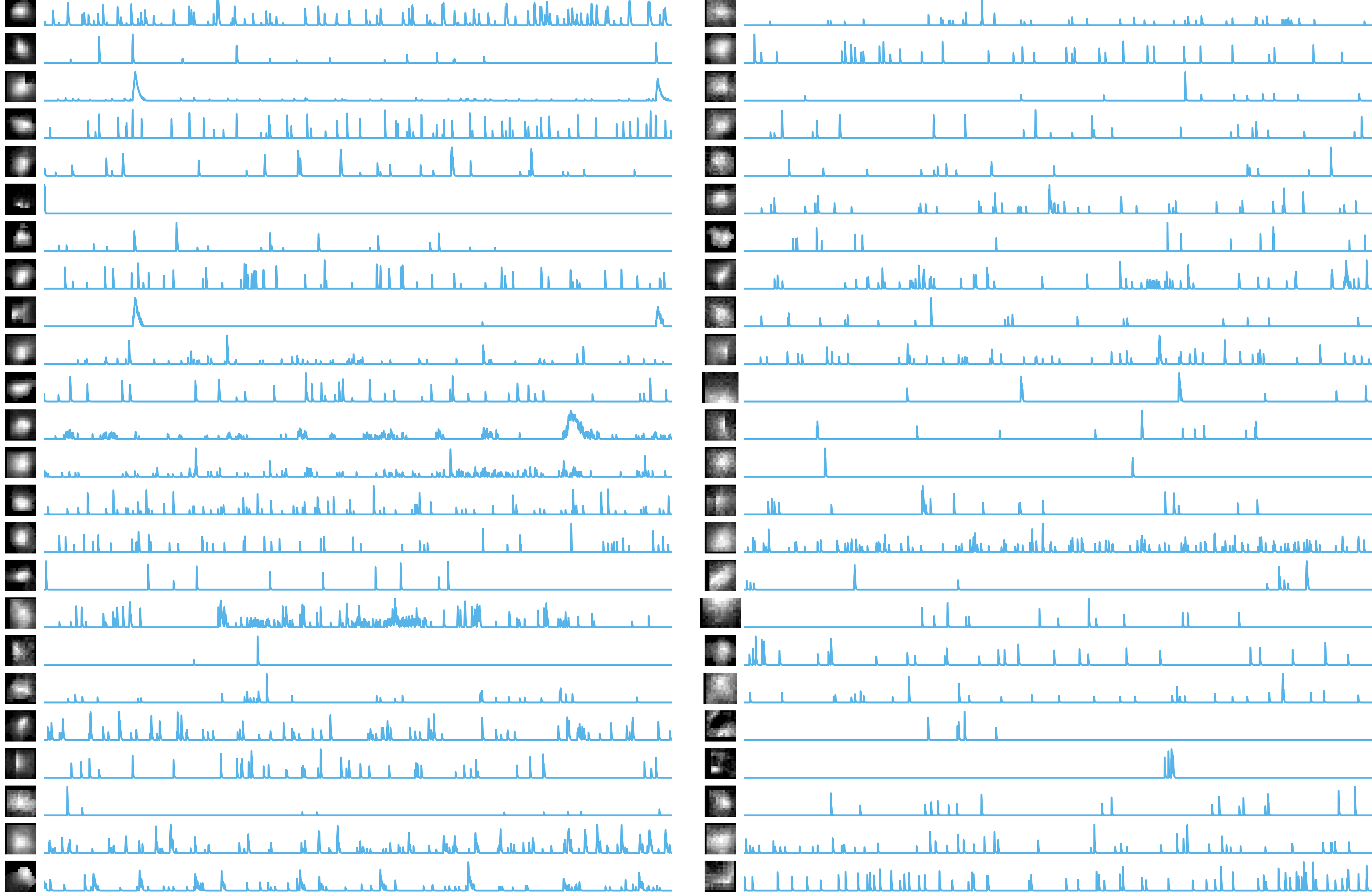

Supplement: S2 Fig — (TIF) [file pcbi.1008565.s003.tif]

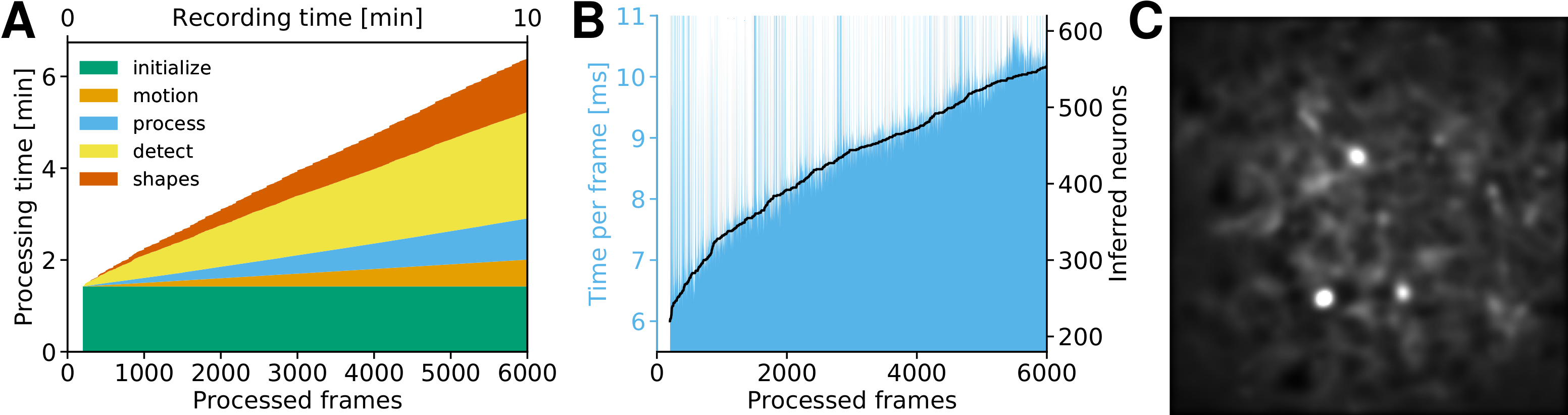

Supplement: S3 Fig — Analogous plots to Fig 7A and 7B, but using the energy for each pixel of the residual buffer to create the summary image instead of the Corr*PNR summary image (see Methods). (TIF) [file pcbi.1008565.s004.tif]

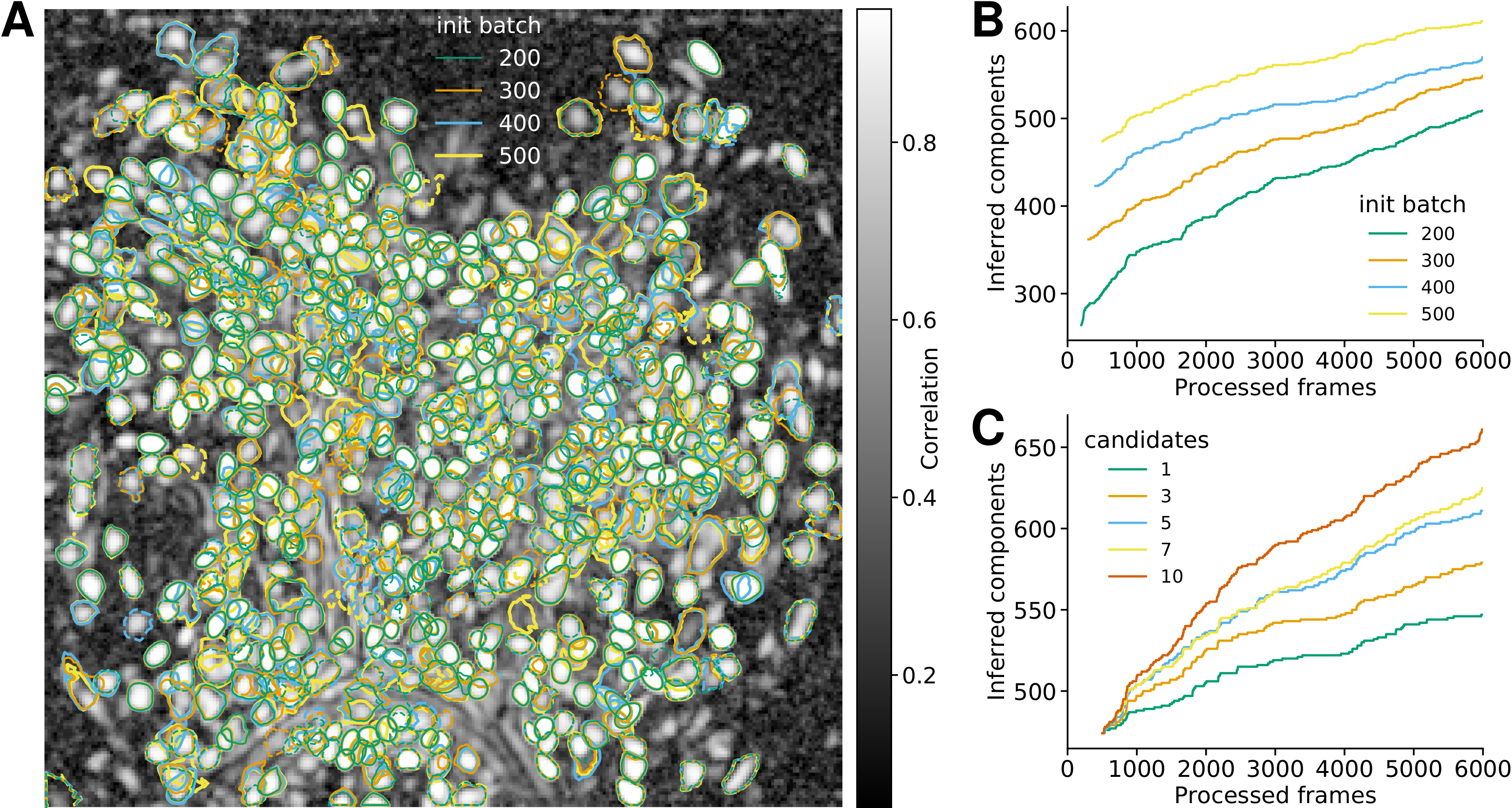

Supplement: S4 Fig — (A) Detected components for different sizes of the initial batch without adjusting other parameters. Components detected in the initial batch are shown as solid contours, those detected during online processing as dashed contours. (B) Number of detected components for the initial batch sizes considered in (A). (C) Number of detected components for a varying number of candidate components. (TIF) [file pcbi.1008565.s005.tif]

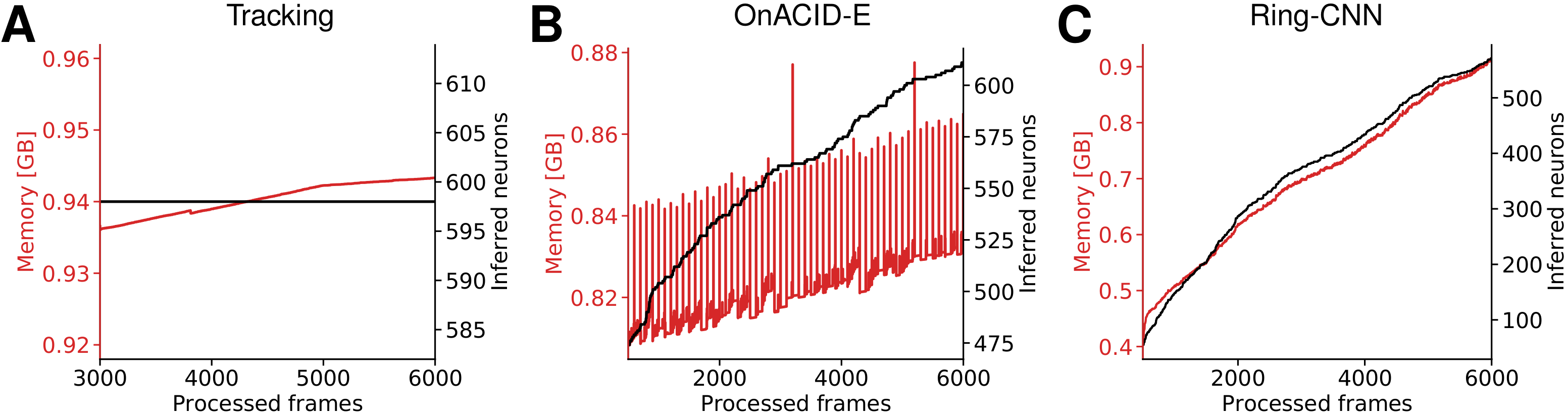

Supplement: S5 Fig — Memory and number of neurons as function of processed frames for (A) Tracking, (B) OnACID-E, and (C) Ring-CNN + OnACID. (TIF) [file pcbi.1008565.s006.tif]

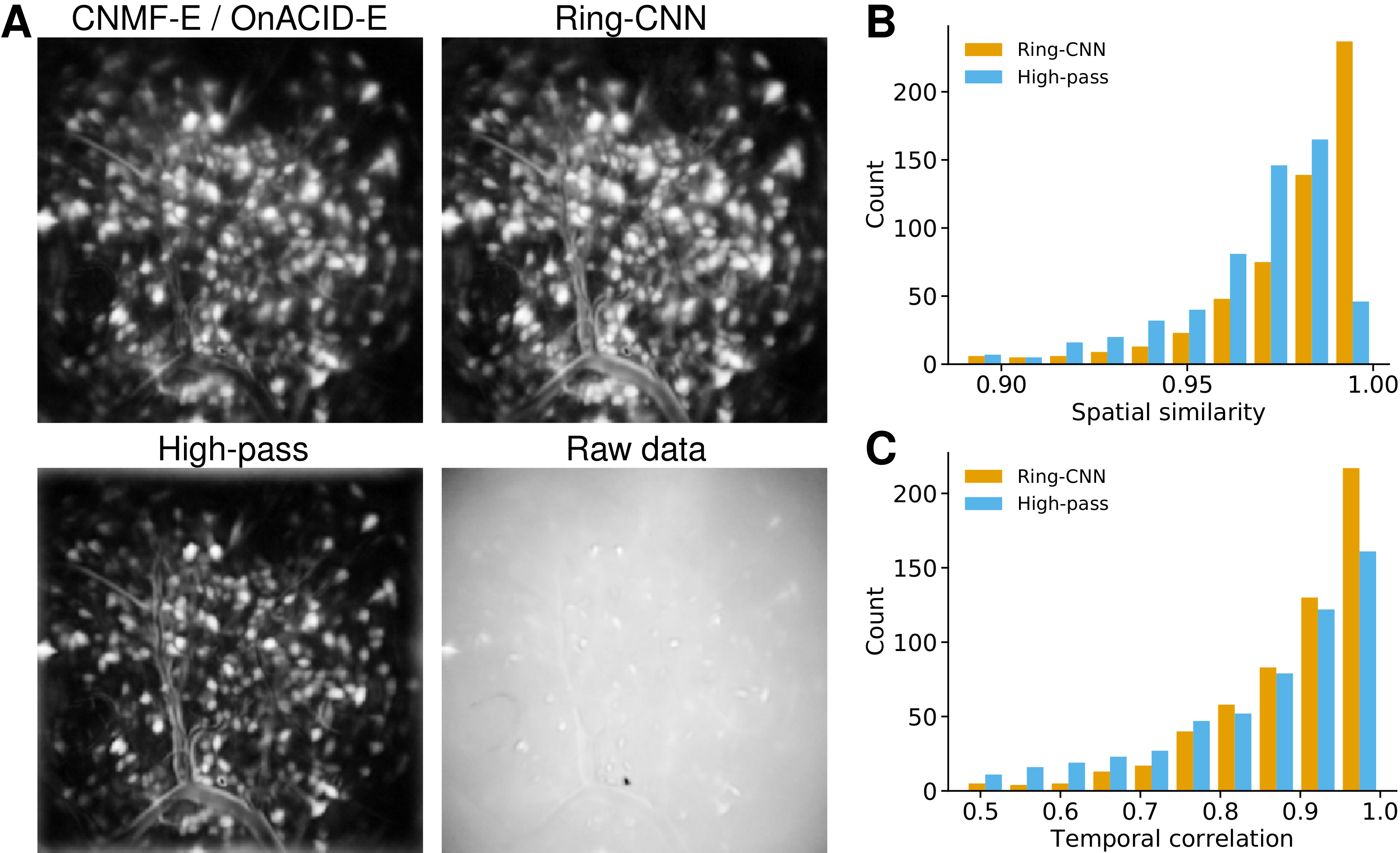

Supplement: S6 Fig — (A) Local cross-correlation images of the background-subtracted video data. (B) Histogram of cosine similarities between inferred neural shapes and the ones obtained with CNMF-E. (C) Histogram of correlations between inferred neural fluorescence traces and the ones obtained with CNMF-E. (TIF) [file pcbi.1008565.s007.tif]
